# Construction of a 3D Bioprinted Microfluidic Platform to Study Breast Cancer Bone Metastasis and Tumor Microenvironmental Influences

**DOI:** 10.1021/acsami.5c15529

**Published:** 2025-10-31

**Authors:** Ting-Wei Chang, Min-Wei Huang, Guo-Chung Dong, I-Chi Lee

**Affiliations:** † Department of Biomedical Engineering and Environmental Sciences, 34881 National Tsing Hua University, No. 101, Section 2, Kuang-Fu Road, Hsinchu 300044, Taiwan (R.O.C); ‡ Institute of Biomedical Engineering and Nanomedicine, 50115 National Health Research Institutes, No.35, Keyan Rd., Zhunan Town, Miaoli 35053, Taiwan (R.O.C)

**Keywords:** tumor spheroid laden bioprinting, breast cancer (BrCa) model, bone metastasis, tumor microenvironment (TME), intravasation, extravasation

## Abstract

Breast cancer (BrCa) frequently metastasizes to bone, severely compromising patient survival. Preventing metastatic spread is, therefore, a crucial therapeutic goal. Tumor matrix stiffness, growth factor gradients, and the bone microenvironment collectively influence cancer progression; however, existing *in vitro* models lack the physiological complexity to capture these interactions. To address this, we developed a 3D biomimetic bone metastasis-on-a-chip platform to recapitulate the key microenvironments involved in BrCa dissemination. This study aimed to develop an *in vitro* metastasis chip that mimics BrCa tumors, the vascular-like region, and the bone microenvironment, enabling investigation of microenvironmental factors during metastasis. Bioinks replicating tumor microenvironments with adjustable stiffness were synthesized, including methacrylated collagen (ColMA) and hyaluronic acid (HAMA). Their chemical, mechanical, and biocompatible properties were optimized. Selected bioinks mimicked BrCa stiffness, allowing tumor spheroid embedding in a two-layered model. The core bioink promoted BrCa proliferation, while the peripheral bioink enhanced stemness and epithelial–mesenchymal transition (EMT), increasing metastatic potential. Furthermore, a 3D bone-like matrix composed of methacrylated gelatin and hydroxyapatite was integrated to simulate the extravasation process, facilitating BrCa cell migration and colonization within the bone-mimicking region. This metastasis-on-a-chip system successfully replicates critical stages of BrCa progression and provides a versatile *in vitro* platform for studying metastatic mechanisms and evaluating potential antimetastatic therapies.

## Graphical abstract

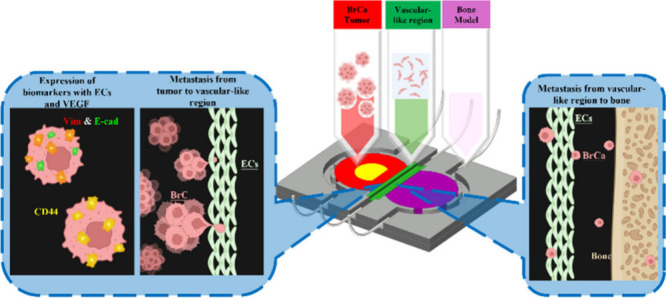

## 1 Introduction

Breast cancer (BrCa) is one of the most common cancers among women, accounting for 10.4% of all cancer cases in females.^1^ In a study involving 264 patients with metastatic breast cancer, 73% were found to have bone metastases.^2^ Targeting cancer cell migration has emerged as a crucial therapeutic goal. However, most current *in vitro* models rely on 2D systems or fail to replicate the complexity of the tumor microenvironment (TME). There is a growing need for preclinical models that can accurately mimic tumor niches and progression as these models could bridge the gap between preclinical studies and clinical trials, facilitating the development of effective therapeutic strategies. Furthermore, such advanced models could support drug discovery in a realistic *in vitro* environment, reducing the reliance on animal experiments and enhancing our understanding of metastatic progression.

The TME is composed of an extracellular matrix (ECM), stromal cells, and soluble factors. The ECM, which includes collagen, laminin, proteoglycans, and glycosaminoglycans, undergoes remodeling through interactions with cancer cells, influencing tumor behavior. Stromal cells, such as fibroblasts and immune cells, further contribute to the TME’s complexity by secreting factors that modify cancer cell dynamics.^3,4^ Increased ECM density, driven by these interactions, enhances the mechanical stiffness of the TME, which has been linked to tumor progression and metastasis.^5,6^ Variations in the tumor site stiffness are closely associated with tumor progression and metastasis. Studies have shown that higher bulk modulus in resected tumors correlates with local recurrence and metastasis, with collagen content directly affecting stiffness.^7^ These mechanical properties are thought to influence bone metastasis in BrCa. Additionally, the ECM, including hyaluronic acid (HA), plays a key role in invasion and therapeutic resistance, both associated with poor prognosis.^8^ HA is a naturally occurring linear, nonimmunogenic polysaccharide. HA is widely present in the ECM of higher animals and plays a critical role in cell–ECM interactions, wound healing, cell differentiation, and cell motility.^9^ In addition, HA is also a key component of stem cell and CSC niches, interacts with CD44, a receptor overexpressed in various tumors, and correlates with tumor progression and metastasis.^10,11^ HA and collagen form the main ECM in the tumor niche, suggesting that mechanically tunable hydrogels composed of methacrylated hyaluronic acid (HAMA), methacrylated collagen (ColMA), and collagen can effectively recapitulate metastatic behaviors. Furthermore, tumors also exhibit significant mechanical heterogeneity. Marija Plodinec et al. reported that normal mammary tissue exhibits a stiffness of approximately 1.13 kPa, whereas benign fibroadenomas range from 1.76 to 5.6 kPa. In breast cancer, the tumor core tends to be softer, while the periphery is stiffer, with stiffness values ranging from 2 to 20 kPa. Hematoxylin and eosin (H&E) staining further revealed a higher cell density in the tumor core, indicating enhanced proliferative activity within this region.^12^

Growth factors in blood vessels are also one of the important TMEs to regulate cancer cell migration. The vascular microenvironment includes factors like fibroblast growth factor (FGF), angiopoietins, platelet-derived growth factor (PDGF), and vascular endothelial growth factor (VEGF), with VEGF-A primarily regulating angiogenesis by promoting endothelial cell proliferation, migration, and tube formation. In hypoxic tumor cores, hypoxia-inducible factor-1α induces VEGF-A expression, driving angiogenesis to supply oxygen and nutrients.^13^ Previous studies have also utilized 3D bioprinting to create multilayered coculture models and stretchable, perfusable vascular lumens, respectively, to better mimic the vascular microenvironment.^14,15^

Current 3D *in vitro* models better replicate heterogeneous tumors and the clinically relevant TME by incorporating cancer cells, ECM, and other factors that 2D models fail to simulate due to the lack of cell interactions and matrix properties. As a result, 90% of drugs tested in 2D models are ineffective in clinical settings, highlighting the need for transitioning to 3D models for more accurate drug testing and tumor modeling.^16^ 3D bioprinting technology is a promising tool for the incorporation of the ECM component, cell–cell interaction, and specific conformation which can provide application of the biomimetic organ construct and toward a disease model.^17^ Bioinks, used in 3D bioprinting, enable cells to grow in a 3D environment with a biological scaffold, supporting cell–ECM interactions and regulating cell behavior to create a complex TME. Several literatures have used 3D bioprinted techniques to study the microenvironment effect on cancer cell behaviors.^18^ Wang et al. have fabricated a 3D bioprinted construct with BrCa cells and a mesenchymal stem cell layer to study the drug resistance effect.^19^ 3D bioprinted constructs can also provide spatial control on matrix properties to model the ECM mechanic variation and effect on metastatic behaviors.^18^ Among them, the effect of matrix stiffness, vessel diameter, and spatial distribution of biochemical factors have been mediated to mimic the native tumor microenvironment and to study the metastasis effect.^20^ However, there remains a scarcity of efficient 3D models to study the metastasis process from breast tumors to bone *in vitro*. Only few existing models have simulated the process between tumor cells and endothelial cells (ECs) or discuss the interaction between cancer cells and bone cells.^21,22^ Therefore, to reduce the need for animal and human testing and enhance drug prediction efficiency, this study employs 3D *in vitro* models to simulate the BrCa microenvironment and metastasis. Figure 1 illustrates BrCa metastasis via a 3D bioprinted cancer-on-a-chip, depicting the bioink compositions used to simulate three distinct regions: the breast cancer tumor, the vascular-like region, and bone. It also represents the metastatic progression of breast cancer cells from the tumor to the vascular region and subsequently from the vascular region to the bone. Using 3D bioprinting technology, bioinks were combined with tumor spheroids to create a realistic BrCa microenvironment. Additionally, a microfluidic system was integrated to supply nutrients and growth factors. This approach provides a platform for researchers and clinicians that closely mimics the actual BrCa metastasis process.

**Figure 1:**
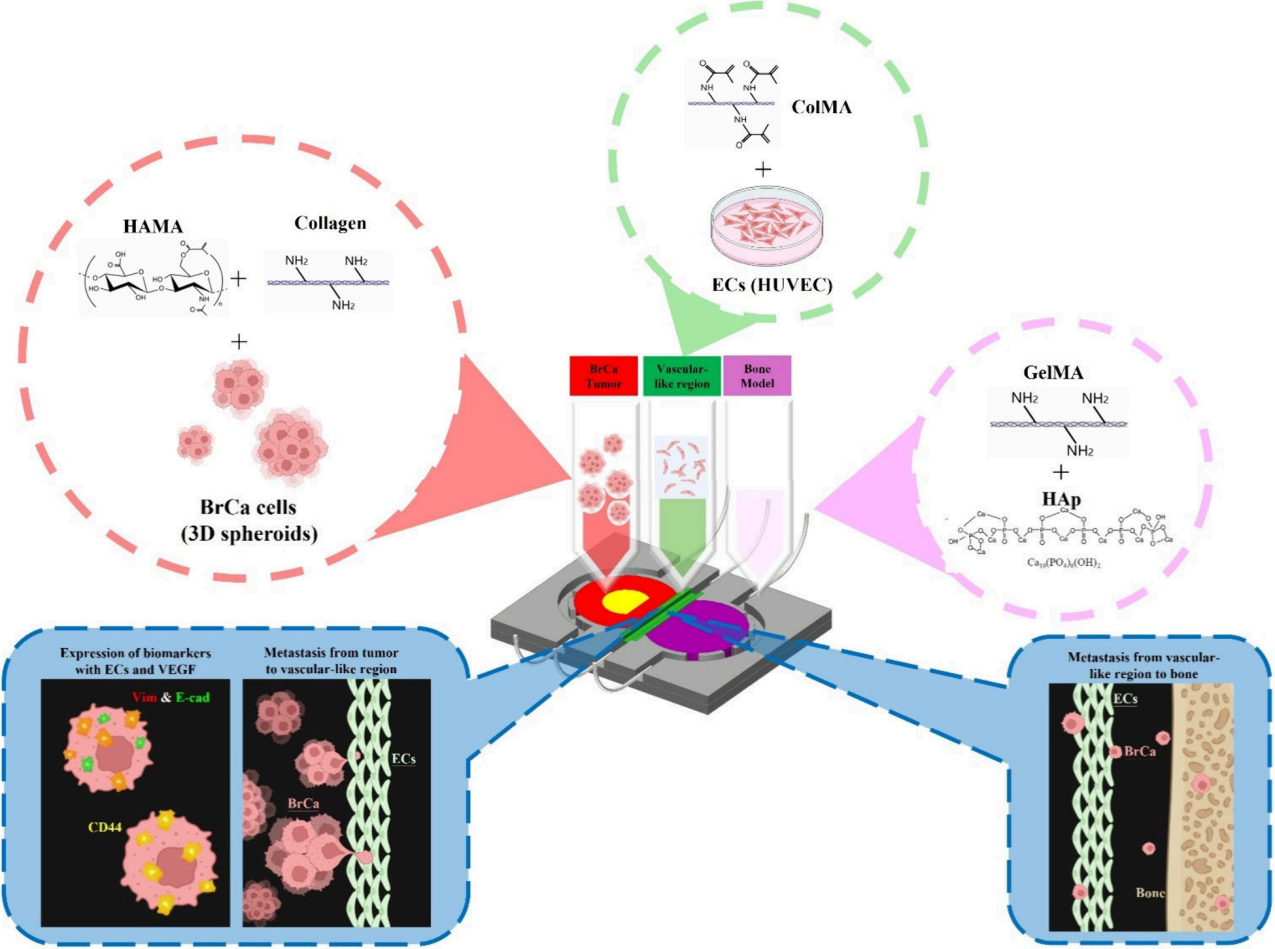
Schematic illustrating breast cancer metastasis via a 3D bioprinted cancer-on-a-chip, depicting the bioink compositions used to simulate three distinct regions: the breast cancer tumor, vascular-like region, and bone. It also represents the metastatic progression of breast cancer cells from the tumor to the vascular region and subsequently from the vascular region to the bone. Some components (e.g., cell spheroids) were created with BioRender.com, and the figure was further modified by the authors.

## 2 Experimental Section

### 2.1 Preparation of ColMA, HAMA, and GelMA Hydrogel

For collagen methacrylate (ColMA) preparation, collagen (Sigma, U.S.A.) was dissolved in deionized water at 37 °C to form a solution. Methacrylic anhydride (MA, Sigma, U.S.A.) was added dropwise, and the mixture was stirred for 24 h while maintaining the pH between 8 and 9. The resulting product was dialyzed in deionized water by using a 12–14 kDa dialysis membrane, with water changes occurring over 3 days. For HAMA preparation, hyaluronic acid (HA, Mw: 300,000, Sigma, U.S.A.) was dissolved in deionized water, reacted with MA, and stirred at 4 °C for 24 h. The solution was dialyzed against deionized water for 7 days, with water changes occurring three times on the first day and twice daily thereafter. The dialyzed solution was then frozen at −20 °C, freeze-dried for 72 h, and stored in a desiccator. For gelatin methacrylate (GelMA) preparation, gelatin was dissolved in PBS at 50 °C while being stirred at 240 rpm until fully dissolved. MA was added dropwise, and the mixture was stirred for 2 h. The reacted solution was then mixed with PBS and stirred for 10 min. The modified GelMA was transferred to 12–14 kDa dialysis membranes and dialyzed in distilled water at 40 °C and 500 rpm for 5 days, with water changes twice daily. After dialysis, the GelMA was frozen at −20 °C for 1 day and freeze-dried for 48 h.

### 2.2 Chemical Structure Analysis by FTIR Analysis and Nuclear Magnetic Resonance (NMR) Spectroscopy

For nuclear magnetic resonance (NMR) analysis, 10 mg of each sample was separately dissolved in 1 mL of D_2_O and transferred to NMR tubes. ^1^H NMR spectra were acquired by using a 600 MHz NMR spectrometer (Avance 500 NMR, Bruker, Germany) at 600 MHz, with D_2_O peaks used as calibration references. Each sample was measured in triplicate. For FTIR analysis, the hydrogels were prefrozen at −80 °C and then lyophilized. The lyophilized samples were mixed with potassium bromide to form pellets. FTIR spectra were recorded in the range 400–4000 cm^–1^ using an FTIR spectrometer (Spectrum Two, PerkinElmer, U.S.A.).

### 2.3 Determine the Concentration and UV Exposure Time for Gelation of a Series of Bioinks in Different Compositions

After freeze-drying, the lyophilized ColMA and HAMA were dissolved in water at varying concentrations, followed by the addition of a photoinitiator (PI, hydroxy-4′-(2-hydroxyethoxy)-2-methylpropiophenone, Irgacure 2959, Sigma, USA) at a concentration of 0.5 wt %. The samples were then exposed to UV light at a wavelength of 365 nm with an intensity of 800 mW/cm^2^ for 30 s.

### 2.4 Mechanical Property, Viscosity, and Rheology Assay of Different Compositions of Hydrogels

The compressive modulus of various bioink compositions was assessed following our previous methodology, using a strain rate of 20% per minute. The modulus was determined from the gradient of the linear region between 0 and 5% strain. For rheological characterization, the material’s response to shear forces was evaluated through continuous flow experiments, with shear rates increasing linearly from 1 to 100 s^–1^. Additionally, oscillatory strain sweeps were conducted to assess the material’s response to incremental strain, spanning from 0.01 to 1000% strain at a frequency of 10 Hz. The bioinks’ shear recovery was also examined by applying and removing shear forces.

### 2.5 SEM Characterization

SEM characterization of different compositions of hydrogel samples was subjected to freeze-drying and cryofractured in liquid nitrogen with a scalpel to expose the fracture surfaces. A field emission scanning electron microscope (FE-SEM) operated at an accelerating voltage of 5 kV was used to investigate sample cross sections and morphology.

### 2.6 Equilibrium Swelling Ratio Measurements

The equilibrium swelling ratio of the hydrogels with different compositions was determined using the gravimetric method. The hydrogels were lyophilized and incubated in PBS solution for 48 h at 37 °C to reach a swelling equilibrium. After the surface moisture was blotted with a Kimwipe, the wet weight of the hydrogels was measured using an electronic balance. The hydrogels were then freeze-dried, and the dry weight was recorded. The equilibrium swelling ratio was calculated based on these measurements.

### 2.7 Cell Viability Assay and Cytotoxicity Assay of a Series of Bioinks

Cell viability was assessed at each time point using the MTT colorimetric assay. After incubating the cells for the specified time, the medium was replaced with MTT solution, and the cells were further incubated for 3 h. The optical density of the formazan solution was measured at 570 nm by using an ELISA plate reader. Cytotoxicity of the cells in different bioinks was evaluated by using a cytotoxicity detection kit to quantify the release of lactate dehydrogenase (LDH) into the culture medium, which serves as an indicator of cell damage. The procedure followed the manufacturer’s instructions, and the results were measured using a multimode microplate reader (BioTek Instruments, USA) at 490 nm absorbance with a reference wavelength of 630 nm. In addition, the viability of cells cultured in different bioinks was evaluated by using a fluorescence-based live and dead assay. After various incubation periods, the procedure was performed according to the manufacturer’s protocol, and images of live (green) and dead (red) cells were captured using a confocal microscope (LSM 800 META, Zeiss, Germany).

### 2.8 Breast Cancer Spheroid Formation and Drug Resistance Property Testing

Two types of breast cancer cell lines, MCF7 and MDA-MB-231, were obtained from the American Type Culture Collection (ATCC). Spheroid formation of breast cancer cells was established based on a modified protocol from our previous study using poly­(vinyl alcohol) (PVA, Sigma, U.S.A.)-coated glass slides. The PVA solution (100 μL per well) was then dispensed into well plates and dried in an oven at 70 °C for 24 h. The PVA-coated plates were sterilized under UV light for 24 h prior to cell seeding. For both cell lines, different PVA coating concentrations and initial seeding densities were tested to evaluate their effects on spheroid formation. The resulting spheroids were observed and analyzed in terms of size and number, and optimal conditions were determined for establishing the final 3D spheroid culture. To assess the drug resistance of doxorubicin (DOX) in breast cancer spheroids, DOX was applied to both 2D monolayer cells and 3D breast cancer spheroids at predetermined concentrations and incubated for 24 or 48 h. After incubation, CCK8 reagent was added, followed by an additional 3 h incubation at 37 °C with 5% CO_2_. Subsequently, 50 μL of supernatant was collected, and the absorbance at 450 nm (O.D. value) was measured using an ELISA reader.

### 2.9 Cell Proliferation Assay and Cell Migration Analysis of BrCa Spheroids in Different Compositions of Bioinks

BrCa spheroids embedded in hydrogels were cultured in medium, and cell proliferation was assessed by CCK8 reagent incubated at 37 °C with 5% CO_2_ for 3 h. Absorbance at 450 nm was measured using an ELISA reader with medium plus CCK8 as the background. The initial proliferation rate was recorded on day 0 as the control, with subsequent measurements taken on days 2, 4, and 6 to monitor proliferation over time. In addition, to prepare for spheroid formation, CellTracker Red and CellTracker Green live cell dyes were added to the cell suspension and incubated at 37 °C with 5% CO_2_ for 30 min. The labeled cells were then centrifuged at 1000 rpm for 5 min; the supernatant was removed; and the cells were seeded onto a PVA-coated well plate to form spheroids. After 3 days of spheroid formation, 3D bioprinting was performed. The BrCa spheroids embedded in different compositions of bioinks were cultured in medium, and cell migration was observed under a microscope on days 4, 7, 10, and 14. Migration distances were quantified by using ImageJ.

### 2.10 Cell Behavior and Immunofluorescence Staining of BrCa Spheroids and ECs in Hydrogels

Immunofluorescence staining was performed on BrCa spheroids and ECs in hydrogels. Samples were rinsed in 1 × PBS for 3 min, repeated three times, and then fixed in 4% paraformaldehyde for 20 min. After fixation, the paraformaldehyde was removed, and the samples were rinsed again in 1× PBS for 3 min, repeated three times. Samples were incubated with primary antibodies (CD44, 1:1000; CD31, 1:100; Vimentin, 1:1000; E-cadherin, 1:1000; Invitrogen, USA) at 4 °C for 72 h. Following incubation, the primary antibody solution was removed, and samples were rinsed three times with 1× PBS for 3 min each. A secondary antibody solution (Goat antimouse IgG H&L, 1:250, Alexa Fluor 488, Abcam, UK; Goat antirabbit IgG H&L, 1:250, Alexa Fluor 488, Abcam, UK) was prepared with DAPI (1:2000, Millipore, USA) and applied to the samples, which were then incubated at room temperature for 3 h. After incubation, the secondary antibody solution was removed, and samples were rinsed three times with 1× PBS for 3 min each. Finally, samples were mounted on microscope slides with coverslips and analyzed for cell marker expression using a confocal microscope.

### 2.11 Chip Design and Cell-Laden 3D Bioprinting

The design and fabrication process of the chip are illustrated in Figure 5(A) and (B). First, the mold was designed by using AutoCAD, as shown in Figure 5(A), with all dimensions explicitly labeled. The mold was then fabricated using an engraving machine to ensure precise dimensional accuracy. Finally, the prepared mold was used to cast the chip with PDMS, as depicted in Figure 5(B). Subsequently, the preformulated bioink was mixed with cells and 3D bioprinted into distinct regions of the chip, as illustrated in Figure 5(C). The extrusion parameters were optimized using Ultimaker Cura based on the nozzle diameter (22 G, ∼0.413 mm), and the corresponding gcode was generated for printing. A single-layer grid pattern was fabricated using a 3D bioprinter and immediately photo-cross-linked with UV light for 30 s to ensure structural stability. The void areas within each 2 × 2 square of the printed grid were quantified using ImageJ, and the printability was calculated according to eq 1:

1 $$\mathrm{Relative Area}\;\left(\%\right)=\frac{\mathrm{The average area of one unit of bioinks}\;\left(n=4\right)}{\mathrm{The average area of one unit of toothpaste}\;\left(n=4\right)}$$

A higher relative area indicates better print fidelity compared with the reference material.

### 2.12 Permeability of the EC-Laden Bioink Barrier

For the permeability assay, SVEC cells were employed in place of HUVECs to establish the endothelial barrier. SVEC4–10 was purchased from the Bioresource Collection and Research Center (BCRC, Taiwan). ECs were encapsulated in 1 wt % ColMA bioink at a density of 2 × 10^6^ cells/50 μL and subsequently printed onto a chip to construct an EC–hydrogel barrier, as illustrated in Figure 5(D). Fluorescein isothiocyanate-dextran (average molecular weight 60,000–76,000) was prepared at a concentration of 1 mg/mL and applied to the left side of the EC–hydrogel barrier. The diffusion of dextran across the endothelial barrier was monitored every 30 min using a fluorescence microscope, and quantitative analysis was performed with ImageJ software. The permeability was calculated according to eq 2, where *I* represents the fluorescence intensity of dextran, and *A* denotes the flow cross-sectional area (2680 × 1000 μm^2^):

2 $$\mathrm{Permeability}=\frac{I_{\mathrm{final}}/I_{\mathrm{initial}}}{t_{\mathrm{final}}-t_{\mathrm{initial}}}A$$

### 2.13 Statistics and Graphical Presentations

In this study, all data are expressed as the mean ± standard deviation (SD) from independent experimental replicates, with a minimum sample size of *n* ≥ 3. Statistical analyses were conducted using Student’s *t* test and two-tailed tests to assess significance. Data visualization and analysis were performed using Excel, GraphPad Prism, TopSpin, and ZEISS ZEN lite, while the chip mold design was created with AutoCAD. Quantitative analyses were carried out using ImageJ software. Statistical significance is denoted by asterisks as follows: *p < 0.05, **p < 0.01, ***p < 0.005, and ****p < 0.001.

## 3 Results and Discussion

### 3.1 Characterization Analysis of HAMA and ColMA

NMR spectroscopy is a powerful analytical technique that is used to analyze molecular structures. Specifically, the ^1^H NMR spectrum examines the nuclear magnetic resonance of ^1^H nuclei in molecules, enabling structural identification and quantification. Figure S1­(A) illustrates the chemical reaction for synthesizing HAMA, where HA is modified with MA to introduce functional groups. In this reaction, MA substitutes for the methyl groups on HA, resulting in the formation of HAMA. Figure S1­(B) shows the ^1^H NMR spectrum of HA, where the signal for methyl protons appears at δ = 1.95 ppm.^23^ In the ^1^H NMR spectrum of HAMA, the signal at δ = 1.95 ppm decreases, indicating a reduction in methyl proton content compared to HA, consistent with substitution by methacrylate. Additionally, new signals corresponding to the proton groups of methacrylate CH_2_=CH­(CH_3_) are observed at δ = 5.7 and 6.1 ppm, confirming the successful incorporation of methacrylate into HA. The degree of substitution (DS) of HAMA was quantified by analyzing the ^1^H NMR spectra. Specifically, the DS was obtained from the ratio between the integrated area of the methacrylate proton peaks and that of the methyl protons of HA. Through this comparison, the DS of HAMA was determined to be approximately 31.8%. Type I collagen consists of a triple-helix structure with a primary amino acid sequence of Gly-X-Y, where X and Y are predominantly proline and hydroxyproline but may include other amino acids such as lysine, glutamine, and asparagine.^24^ Figure S1 illustrates the chemical modification of collagen to form ColMA, where MA reacts with amino groups on lysine, glutamine, and asparagine, introducing methacrylate functional groups into the collagen structure.

In Figure S1­(E), the ^1^H NMR spectrum shows a noticeable decrease in the signal corresponding to the ε-methylene protons of lysine at δ = 2.9 ppm after methacrylation, accompanied by the appearance of new vinyl proton peaks at δ = 5.85 and 6.30 ppm, which are attributed to the CH_2_=CH­(CH_3_) groups of the methacrylate moieties. These spectral changes confirm the successful conjugation of methacrylate groups to the collagen amino residues, resulting in the formation of ColMA. Similarly, the DS of ColMA was quantified as 97.86% based on the lysine methylene integral (2.8–2.9 ppm).

Additionally, we utilized FTIR to further identify the functional groups of HA, HAMA, collagen, and ColMA. As shown in Figure S1­(C), both HA and HAMA exhibit a characteristic stretching peak of the CO group in alcohols at 950 cm^–1^ and peaks corresponding to the glycosidic linkages between d-glucuronic acid and *N*-acetyl-d-glucosamine units at 1050 cm^–1^. Following modification of HA with MA, new peaks appear in the HAMA spectrum at 1643 cm^–1^ and 1731 cm^–1^, corresponding to C=C and C=O stretching, respectively.^25^ These spectral changes confirm the successful modification of HA by MA. In addition, as shown in Figure S1­(F), distinct peaks at 960 cm^–1^ and 1100 cm^–1^ are observed, corresponding to the C=C group of MA and the C–N bond formed between collagen and the MA group, respectively. These peaks confirm the successful modification of collagen with MA.^26^ Furthermore, the characteristic peaks at 1656 cm^–1^, 1548 cm^–1^, and 1240 cm^–1^, corresponding to amide I, amide II, and amide III of collagen, respectively, remain intact. This indicates that the modification process does not disrupt or affect the triple-helix structure of collagen.^27^

### 3.2 Gelation and Mechanical Properties of a Series of HAMA/ColMA/Collagen Bioinks

The synthesized HAMA and ColMA underwent photopolymerization of their MA groups under UV light, facilitated by a PI. Upon UV exposure, the PI absorbed light energy and generated free radicals, which initiated cross-linking by reacting with MA double bonds, resulting in hydrogel formation.^26^ HAMA at concentrations of 1.5, 2, and 3 wt % was combined with varying amounts of collagen to mimic the ECM components of the BrCa microenvironment. UV exposure was applied at 100% intensity for 30 s. As shown in Figure 2(A), all groups successfully formed stable 3D hydrogels, with 3 wt % HAMA representing the solubility limit. Although collagen exhibited fewer solubility constraints, its application was limited due to high costs. Subsequently, pure ColMA hydrogels at 1 and 1.5 wt %, as well as dual-cross-linked hydrogels incorporating ColMA with HAMA and collagen, were evaluated. All ColMA-based hydrogels successfully formed stable 3D structures with concentrations constrained by the cost of raw materials. The ColMA/HAMA/collagen dual-cross-linked hydrogels also demonstrated structural stability, underscoring their potential for modeling the BrCa ECM.

**Figure 2:**
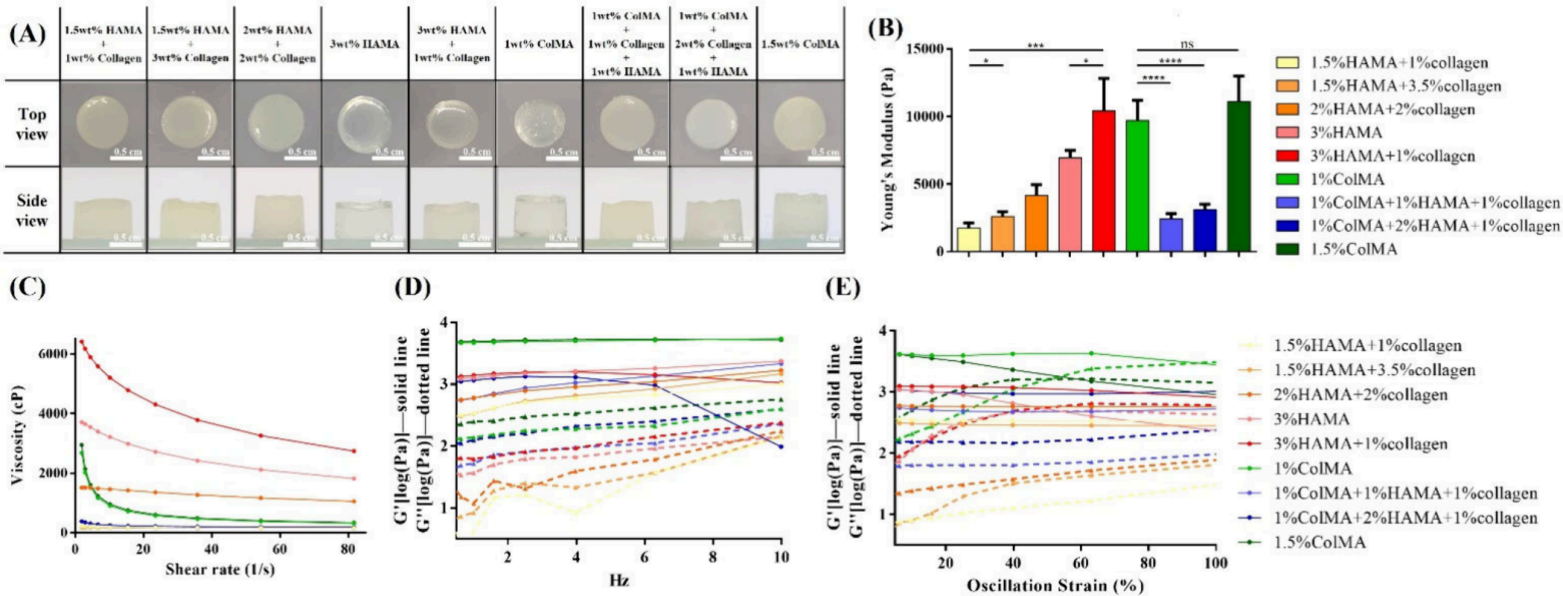
Characterization of HAMA/ColMA/collagen hydrogels. (A) Top and side views of the HAMA/collagen/ColMA hydrogel morphology. (B) Young’s modulus, (C) flow curves, (D) frequency sweep, and (E) strain sweep of HAMA/ColMA/collagen hydrogels. Statistical significance is denoted by asterisks as follows: *p < 0.05, **p < 0.01, ***p < 0.005, and ****p < 0.001.

The stiffness of hydrogels was evaluated through compression testing to determine their Young’s modulus. Previous studies have shown that the tumor core of BrCa patients exhibits a lower Young’s modulus (0–2 kPa), while the tumor periphery has higher values (5–15 kPa).^12^ Based on these findings, hydrogels were analyzed to identify suitable stiffness ranges for mimicking these microenvironments. The compositions of all hydrogel formulations, including the final selections for the tumor core and periphery, are summarized in Table S1, providing a comprehensive overview of polymer combinations and concentrations. As shown in Figure 2(B), increasing the HAMA content resulted in statistically significant increases in hydrogel stiffness, and at a fixed HAMA concentration, the addition of collagen further produced a significant modulus increase. ColMA achieved stiffness comparable to 3 wt % HAMA + 1 wt % collagen with a lower concentration, though additional ColMA did not significantly enhance stiffness. Interestingly, when HAMA and ColMA were combined, all mixed groups consistently showed a statistically significant decrease in Young’s modulus compared to the individual formulations of 2 wt % HAMA or 1 wt % ColMA. This was likely due to the formation of a heterogeneous network during cross-linking, where steric hindrance and limited free radical diffusion led to uneven polymer structures.

### 3.3 Rheology of HAMA/ColMA/Collagen Hydrogels

To evaluate the shear-thinning behavior of the bioinks during extrusion, an analysis was performed on nine formulations to determine their ability to reduce viscosity under shear strain and facilitate smooth ejection through a needle. Figure 2(C) demonstrates the flow curves of the different bioinks, where most formulations exhibited shear-thinning behavior, characterized by a decrease in viscosity with increasing shear strain. Bioinks with higher initial viscosity displayed a more pronounced reduction in viscosity under shear strain, whereas those with lower initial viscosity showed a less significant decrease. Furthermore, the rheological properties of the hydrogels were assessed using a rheometer to measure their storage modulus (*G*′) and loss modulus (*G*″). *G*′ reflects the material’s ability to store energy via elastic deformation, while *G*″ indicates energy dissipation through viscous deformation. Figure 2(D) shows the frequency sweep results (0.01–10 Hz) at 0.5% strain. For most hydrogels, *G*′ exceeded *G*″, signifying elastic-dominant behavior, and both moduli increased with angular frequency, indicating enhanced energy storage and dissipation. However, for the 1 wt % ColMA/2 wt % HAMA/1 wt % collagen hydrogel, *G*′ decreased and fell below *G*′′ at frequencies above 6 Hz, suggesting structural failure and transition to liquid-like behavior. Higher HAMA content resulted in increased *G*′ and *G*′′, while collagen had a minimal effect. ColMA produced hydrogels with comparable moduli at lower concentrations, but further increases in ColMA had a limited impact. A strain sweep (0.01–100% strain, 1 Hz) was conducted to evaluate structural stability under deformation (Figure 2(E)). Most hydrogels maintained an elastic solid state (*G*′ > *G*″) at strains below 50%, but hydrogels with higher *G*′ and *G*′′, such as 3 wt % HAMA, 1 wt % ColMA, and 1.5 wt % ColMA, showed structural breakdown at higher strains, resulting in *G*′ falling below *G*″. In contrast, hydrogels containing unmodified collagen retained *G*′ > *G*′′ even under high strain, preserving elasticity and preventing rupture. Notably, 1 wt % ColMA exhibited superior viscoelastic properties compared to 1.5 wt % ColMA, maintaining higher *G*′ and *G*′′ under strain. Based on these results, 1.5 wt % HAMA/1 wt % collagen (1.73 ± 0.35 kPa) was selected to mimic the BrCa tumor core stiffness, while 3 wt % HAMA/1 wt % collagen (10.4 ± 2.08 kPa) was chosen to represent the BrCa tumor periphery. Additionally, 1 wt % ColMA (9.71 ± 1.3 kPa) was selected for vascular-like model, given its elasticity and alignment with collagen-rich regions. These three hydrogels were selected for subsequent material analyses.

### 3.4 Characterization of HAMA/ColMA/Collagen Hydrogels

Figure 3(A) illustrates the printability of HAMA/ColMA/collagen hydrogels with toothpaste used as a control. Printability was assessed by analyzing the unfilled areas within a 2 × 2 grid using ImageJ. The average unfilled area for each hydrogel group was normalized to the control, with results expressed as a relative percentage. A value closer to 100% indicates that the bioink closely matches the control’s ability to maintain defined edges and shapes without collapse or spreading. Figure 3(B) presents the quantified printability results. Increasing HAMA content significantly improved bioink printability, with the 3 wt % HAMA/1 wt % collagen hydrogel achieving near-perfect shape retention comparable to the control. In contrast, the 1.5 wt % HAMA/1 wt % collagen hydrogel demonstrated poor shape maintenance. Similarly, while 1 wt % ColMA displayed moderate shape retention, its printability was not as robust. These findings highlight the importance of the HAMA concentration in optimizing bioink stability and precision during extrusion-based printing. In the context of our metastasis-on-a-chip model, however, perfect printability is not the sole determining factor. Since each hydrogel is deposited within geometrically confined regions of the microfluidic chip, minor deviations in shape fidelity have a limited impact. Therefore, the final hydrogel selection prioritized physiologically relevant stiffness while ensuring sufficient printability for reliable deposition. Figure 3(C) shows the swelling behavior of the HAMA/ColMA/collagen hydrogels. HAMA-containing formulations exhibited markedly higher swelling ratios, which plateaued as water absorption reached equilibrium, reflecting the strong hydrophilicity of HAMA. Although swelling tended to increase with HAMA content, no significant difference was observed between the 1.5% HAMA + 1% collagen and 3% HAMA + 1% collagen groups, suggesting that water uptake reaches equilibrium at higher HAMA concentrations. Both HAMA groups, however, displayed significantly greater swelling than the 1% ColMA hydrogel. ColMA-based hydrogels exhibited minimal swelling, consistent with their dense, compact structure and lower hydrophilicity. Collagen, despite containing some hydrophilic domains, contributed limited water uptake due to its overall hydrophobicity and the formation of a more rigid, cross-linked network. Figure 3(D) presents SEM images of the hydrogels, revealing differences in the pore size and density across formulations. ColMA hydrogels show a compact, low-porosity architecture, which limits water retention and aligns with their low swelling ratios. In contrast, HAMA-based hydrogels form more open, porous networks that facilitate water absorption, consistent with their higher swelling. Taken together, these results demonstrate that hydrogel swelling behavior and stiffness are strongly influenced by polymer composition, network structure, and cross-linking density. Dense or tightly cross-linked structures, such as in 1 wt % ColMA, limit water retention but still result in higher Young’s modulus due to the compact polymer network. In contrast, 3 wt % HAMA/1 wt % collagen forms a more open network that allows higher water uptake while also maintaining substantial stiffness. These observations highlight that hydrogel microstructure governs both swelling behavior and mechanical properties, but the relationships depend on the specific polymer composition and network architecture.

**Figure 3:**
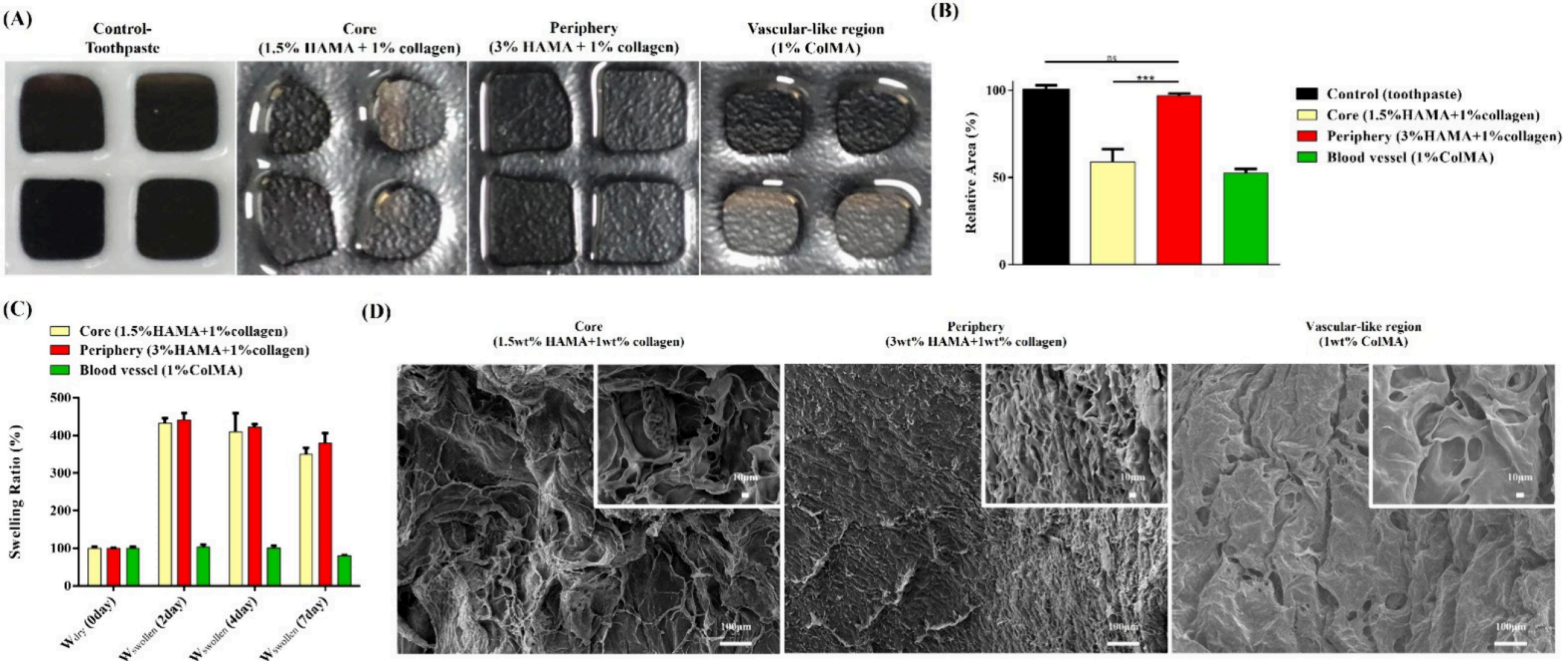
Characterization of HAMA/ColMA/collagen hydrogels. (A) The printing results of HAMA/ColMA/collagen bioink solution. (B) Relative area excluding bioink solution. (C) Swelling ratio of HAMA/ColMA/collagen hydrogels. (D) SEM images of HAMA/ColMA/collagen hydrogels. Statistical significance is denoted by asterisks as follows: *p < 0.05, **p < 0.01, ***p < 0.005, and ****p < 0.001.

### 3.5 Biocompatibility of HAMA/ColMA/Collagen Bioinks

The biocompatibility of three bioink compositions was evaluated using live/dead staining, LDH assay, and MTT assay, as shown in Figure S2. Fluorescence microscopy images in Figure S2 illustrate that L929 cells cultured in medium exposed to the three kinds of bioinks predominantly exhibit green fluorescence, indicating high viability. Quantitative analysis in Figure S2­(B) confirms nearly 100% cell viability across all groups. The LDH assay, which measures cell death via LDH release from damaged cells, showed in Figure S2­(C) that absorbance values for the hydrogel-treated groups were comparable to those of the control, suggesting minimal cytotoxicity. Similarly, the MTT assay, which quantifies metabolic activity through the formation of formazan crystals by live cells, revealed in Figure S2­(D) that absorbance values for three bioinks were near those of the control, confirming high cell viability. These results demonstrate that the three bioinks exhibit excellent biocompatibility with negligible cytotoxicity, making them suitable for biological applications.

### 3.6 Spheroid Formation and Chemotherapy Drug Response in 2D Cultures vs 3D Tumor Spheroids

Tumor spheroids of MCF-7 and MDA-MB-231 cells were generated by using a PVA coating method. For MCF-7 cells, seeding densities of 3 × 10^4^ and 5 × 10^4^ cells per well were tested with PVA concentrations of 0.5, 1.5, and 2.5 wt %. Spheroid formation was monitored daily under a microscope (Figure S3­(A), days 1∼3). Lower PVA coating concentrations (0.5 wt %) produced a higher number of smaller spheroids (<200 μm), whereas 1.5 wt % PVA generated fewer but larger spheroids (∼300 μm). At 2.5 wt %, cells aggregated into large, irregular spheroids exceeding 500 μm, which were not suitable for downstream experiments. Based on these results, 1.5 wt % PVA with a seeding density of 5 × 10^4^ cells per well was selected for subsequent studies, yielding a homogeneous population of spheroids within the physiologically relevant size range (200–400 μm).

For MDA-MB-231 cells, a seeding density of 5 × 10^4^ cells per well was applied across PVA coating concentrations ranging from 0.5 to 2.5 wt %. Spheroid formation was observed at days 1, 3, and 4 (Figure S3­(B)). Similar to MCF-7, lower PVA concentrations produced smaller spheroids, while higher concentrations decreased the spheroid number but increased their size. Spheroids formed under 2 wt % PVA reached an average diameter of ∼200 μm and were chosen for subsequent DOX treatment experiments. Quantitative assessment of spheroid number and diameter after prolonged culture is presented in Figure S4. Representative images of MCF-7 spheroids at day 3 with 1.5 wt % PVA and MDA-MB-231 spheroids at day 4 with 2 wt % PVA are shown in Figures S4­(A) and S4­(B), respectively. The number of spheroids per well is quantified in Figures S4­(C) and S4­(D), and the corresponding spheroid diameters are shown in Figures S4­(E) and S4­(F). Overall, although both cell lines were seeded at the same density (5 × 10^4^ cells/well), the optimal PVA concentration differed (1.5 wt % for MCF-7 and 2 wt % for MDA-MB-231) to generate spheroids within the target size range of 200∼400 μm, ensuring physiological relevance and consistency for downstream drug testing and 3D bioprinting applications. As shown in Figure S4­(G) and S4­(H), both MCF-7 and MDA-MB-231 cells exhibited higher viability in 3D spheroids compared to their 2D counterparts following Doxorubicin treatment, reflecting enhanced drug resistance in the 3D models. In most tested concentrations, the differences between 2D and 3D cultures were statistically significant (*p* < 0.05). Specifically, 2D MCF-7 cells exhibited an IC50 of 25 μM DOX after 48 h and 50 μM after 24 h. In contrast, 3D MCF-7 spheroids did not reach IC50 even at 100 μM DOX after 48 h. Notably, significant differences were observed between 2D and 3D cultures at 25 μM (48 h, ****p < 0.001) and 50 μM (48 h, ***p < 0.005), confirming the enhanced drug resistance of the 3D spheroids. Similarly, in MDA-MB-231 cells, IC50 was achieved in 2D cultures within 48 h, whereas 3D spheroids remained resistant even at the highest tested DOX concentration. Notably, MCF-7 spheroids displayed an initial reduction in proliferation after 24 h of treatment but recovered by 48 h, suggesting that their TME facilitates drug resistance and sustained growth. Conversely, MDA-MB-231 spheroids exhibited a continuous decline in proliferation over 48 h, indicating that their smaller diameter (∼200 μm) may limit the establishment of robust TME gradients, leading to comparatively weaker drug resistance than MCF-7 spheroids (∼300 μm). Preformed spheroids preserve native cell–cell interactions, extracellular matrix organization, and nutrient gradients. Embedding these spheroids before bioprinting maintains their architecture, enabling uniform spheroid size and reproducible, physiologically relevant drug testing outcomes. These findings underscore the critical role of 3D tumor architecture and the TME in recapitulating clinical drug resistance and highlight the limitations of conventional 2D cultures in assessing chemotherapy efficacy.

### 3.7 Proliferation, Migration, and EMT Ability of BrCa Cells in Core and Peripheral Bioinks

To investigate whether BrCa cells in the tumor core exhibit higher proliferative potential, we performed CCK-8 assays to assess the proliferation of MDA-MB-231 and MCF-7 cells cultured in bioinks mimicking the tumor core and peripheral regions on days 2, 4, and 6. As shown in Figure 4(A), with day 0 values as the control group, the results revealed that BrCa cells in core hydrogels, which are softer and have a lower HA content, exhibited greater proliferation compared to those cultured in peripheral hydrogels. This finding supports the notion that BrCa cells within the tumor core exhibit enhanced proliferative capacity, consistent with the findings of Chun Liu et al. in a previous study.^28^ However, the initial higher proliferation observed in the core region compared to the periphery at day 2 gradually diminishes by day 6. This convergence may result from spatial limitations within the hydrogel, which restrict overall spheroid expansion, and from diffusion limitations of nutrients and oxygen that affect both regions. Such microenvironmental constraints likely slow cell growth, leading to comparable proliferation rates at later time points. Additionally, while Lekha Shah et al. demonstrated that MCF-7 cells generally have a higher proliferation rate than MDA-MB-231 cells due to their epithelial nature, no significant differences in proliferation rates between these two cell lines were observed in this study, possibly due to the short culture duration, which may not have been sufficient to distinguish the proliferation patterns of the two cell types.^29^

**Figure 4:**
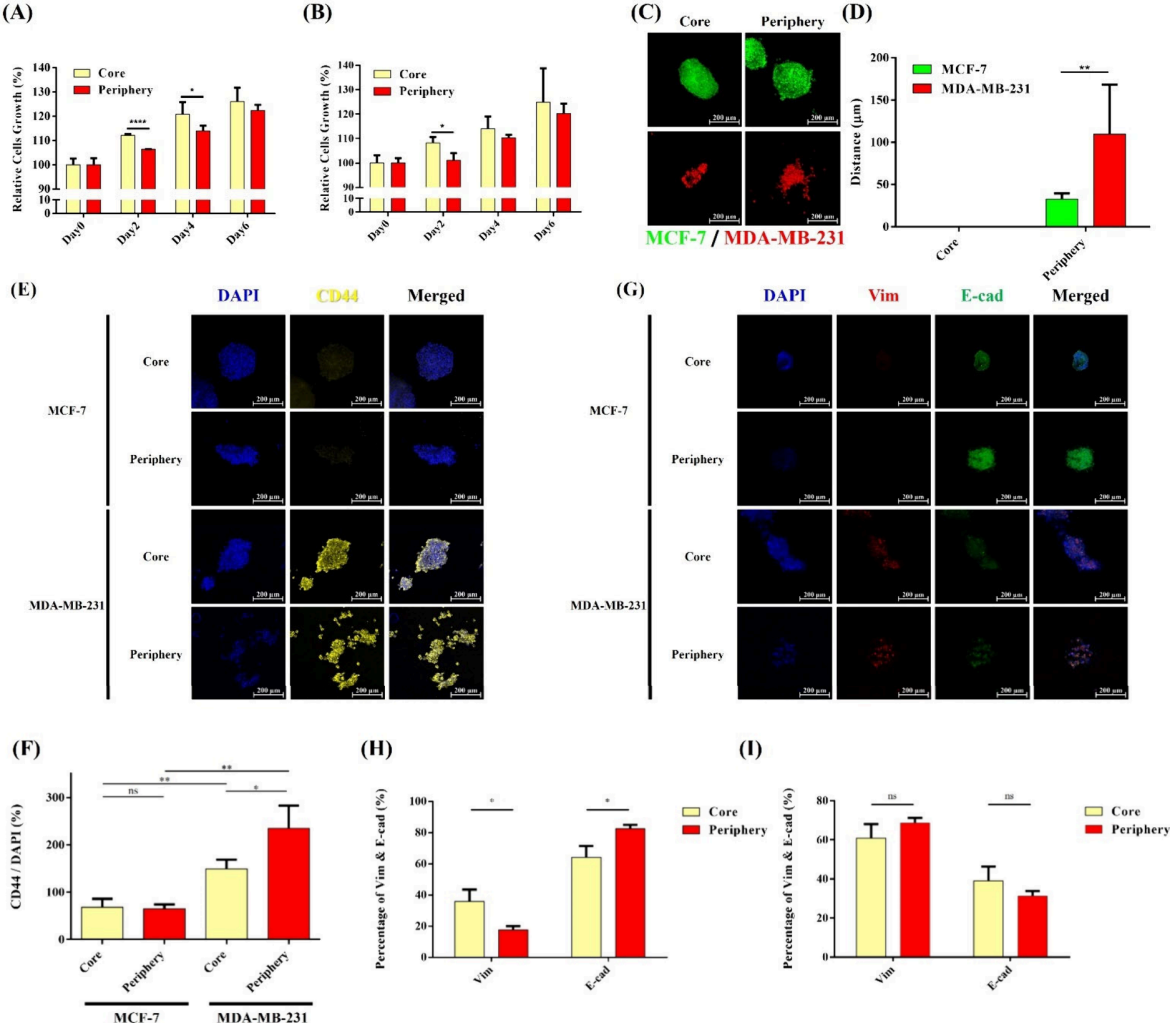
Proliferation of (A) MCF-7 spheroids and (B) MDA-MB-231 spheroids in core and periphery hydrogels at day 0, 2, 4, and 6 (by CCK8 kit). (C) Cell migration and (D) quantifying the migration distance of MCF-7 spheroids and MDA-MB-231 cells in core and periphery hydrogels at day 14. (E) The CD44 expression of immunofluorescence images of MCF-7 and MDA MB-231 in core and periphery hydrogels. (F) The CD44 quantification of MCF-7 and MDA MB-231 in core and periphery hydrogels. (G) The vimentin and E-cadherin expression of immunofluorescence images of MCF-7 and MDA MB-231 in core and periphery hydrogels. (H) The vimentin and E-cadherin quantification of MCF-7 in core and periphery hydrogels at day 7. (i) The vimentin and E-cadherin quantification of MDA MB-231 in core and periphery hydrogels at day 7. Statistical significance is denoted by asterisks as follows: *p < 0.05, **p < 0.01, ***p < 0.005, and ****p < 0.001.

Previous studies have demonstrated that BrCa cells in the tumor periphery exhibit enhanced migratory capabilities. To investigate this, CellTracker Red and CellTracker Green were used to track the migration of MCF-7 and MDA-MB-231 cells cultured in bioinks mimicking the tumor core and peripheral regions. Figure 4(C) shows fluorescence images of MCF-7 and MDA-MB-231 spheroids cultured in core and peripheral hydrogels over 14 days of incubation. Detailed images and quantification of cell migration at days 4, 7, 10, and 14 are shown in Figure S6, providing a comprehensive view of the temporal progression of MCF-7 and MDA-MB-231 spheroid migration. It is visually apparent that both cell types exhibited higher migration in the peripheral hydrogel formulations, indicating that BrCa cells under these conditions possess enhanced migratory capacity. Figure 4(C) quantifies the migration distances of the two cell types in peripheral hydrogels, showing that MDA-MB-231 cells migrated farther than MCF-7 cells. This supports the notion that MDA-MB-231 cells, being more mesenchymal and aggressive, exhibit greater migratory potential compared with the epithelial MCF-7 cells.

To investigate whether core and peripheral bioinks influence the expression of CD44 and EMT markers, MCF-7 and MDA-MB-231 spheroids were embedded in these hydrogels and cultured for 7 days. Immunofluorescence staining was performed for the observation and quantification. As shown in Figure 4(E), MDA-MB-231 cells exhibited significantly higher CD44 expression compared to MCF-7 cells, confirming that the more aggressive MDA-MB-231 cells express higher levels of CD44, a key marker involved in BrCa cell migration and metastasis. Quantification in Figure 4(F) further supports this, showing that CD44 expression in MDA-MB-231 cells is consistently higher than that in MCF-7 cells, regardless of whether cultured in core or peripheral hydrogels. Furthermore, while there was no significant difference in CD44 expression in MCF-7 cells between the core and peripheral hydrogels, MDA-MB-231 cells displayed greater CD44 expression in peripheral hydrogels. This suggests that the tumor periphery ECM microenvironment may promote upregulation of CD44 in MDA-MB-231 cells, potentially enhancing their migratory and metastatic capabilities.

Figure 4(G) examines the expression levels of vimentin and E-cadherin in MCF-7 and MDA-MB-231 spheroids cultured in core and peripheral hydrogels. As shown in Figures 4(G) and 4(H), MCF-7 cells exhibit E-cadherin expression higher than that of vimentin, indicating a stronger presence of E-cadherin, which promotes epithelial cell adhesion and reduces invasiveness. In contrast, Figures 4(G) and 4(I) show a significantly increased expression of vimentin in MDA-MB-231 cells, with vimentin consistently being more abundant than E-cadherin in both core and peripheral hydrogels. This result confirms that MDA-MB-231 cells exhibit mesenchymal characteristics, typical of invasive breast cancer cells. Although vimentin expression in MDA-MB-231 cells is slightly higher in peripheral hydrogels compared to core hydrogels, the difference is not statistically significant.

### 3.8 Permeability of the EC–Hydrogel Barrier

As previously described, the design, fabrication process of the chip, and 3D bioprinting into different regions of the chip for mimicking three models were illustrated in Figure 5(A)–(C). First, the mold design is created by using AutoCAD, as illustrated in Figure 5(A), with all dimensions explicitly labeled in the image. To investigate whether ECs embedded in hydrogels can establish an EC barrier to simulate BrCa infiltration into the vascular-like region, a dextran solution was used to compare the permeability of hydrogels alone versus EC-laden 3D bioprinting hydrogels. Permeation of dextran was monitored every 30 min using fluorescence microscopy to evaluate the formation and function of the EC barrier in reducing permeability. As illustrated in Figure 5(E), the dextran solution was placed on the left side, while the right side contained hydrogels with or without embedded ECs. Over time, dextran gradually diffused from the left side to the right side. Here, the hydrogel-only group represents the fully permeable control condition, reflecting the absence of a barrier at the initial stage. Quantitative analysis, presented in Figure 5(F), demonstrates that dextran permeability was significantly higher in the hydrogel-only group, indicating a lack of barrier function. In contrast, the group with embedded ECs exhibited a reduced permeability, confirming the successful formation of an EC barrier that effectively limited dextran diffusion.

**Figure 5:**
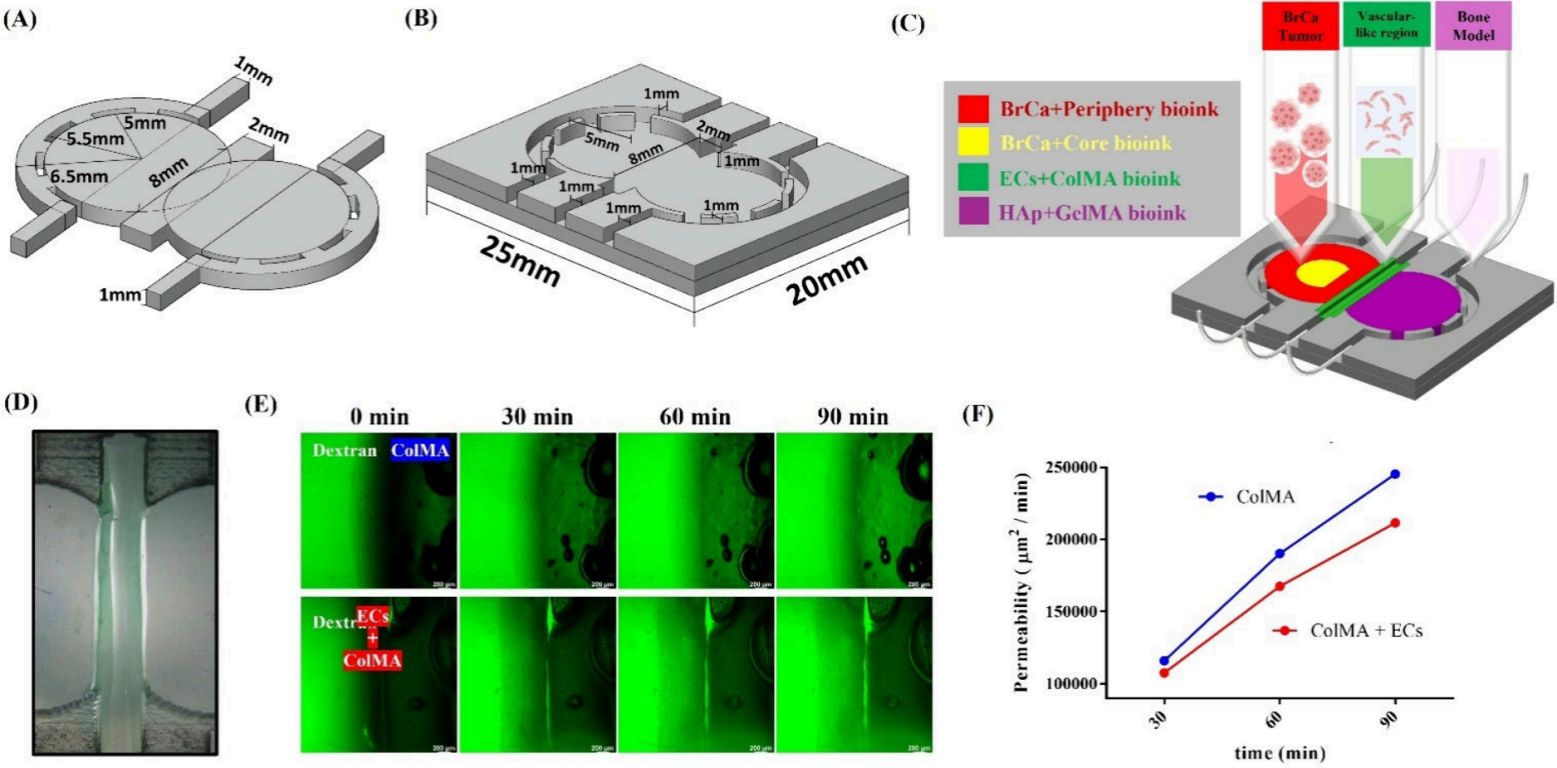
(A) Design mold of the chip. (B) Chip replicated using PDMS molding. (C) Schematic diagram of spheroid-laden 3D bioprinted hydrogel on a chip. (D) Top view of vascular-like model. (E) Images and (F) quantification of comparing permeability with and without the EC barrier (dextran is placed on the left side). Some components (e.g., cell spheroids) were created with BioRender.com, and the figure was further modified by the authors.

### 3.9 Intravasation of BrCa (MDA-MB-231) into a Vascular-Like Region on Cancer-on-a-Chip

To simulate the role of ECs in promoting BrCa invasion and metastasis, HUVEC-laden 3D bioprinting was incorporated into a “BrCa metastasis on-a-chip” model using a microfluidic culture system maintained for 7 days. Figure 6(A) and (B) shows the 3D bioprinting of the tumor region, vascular-like region, and bone region on the chip and schematic diagram of BrCa cells intravasation into ECs on a chip. The expression of CD44 and EMT markers (vimentin, E-cadherin) in the tumor region and CD31 in the vascular region was analyzed to assess HUVECs’ influence on BrCa cell invasion. As shown in Figure 6(C) and (D), the presence of HUVECs did not significantly enhance CD44 expression, likely due to its role as a receptor for HA, which is not modulated by HUVECs. While CD44 is utilized during BrCa extravasation, there is no evidence of its involvement in EC barrier crossing during invasion. However, the addition of VEGF significantly increased CD44 expression and promoted MDA-MB-231 infiltration.

**Figure 6:**
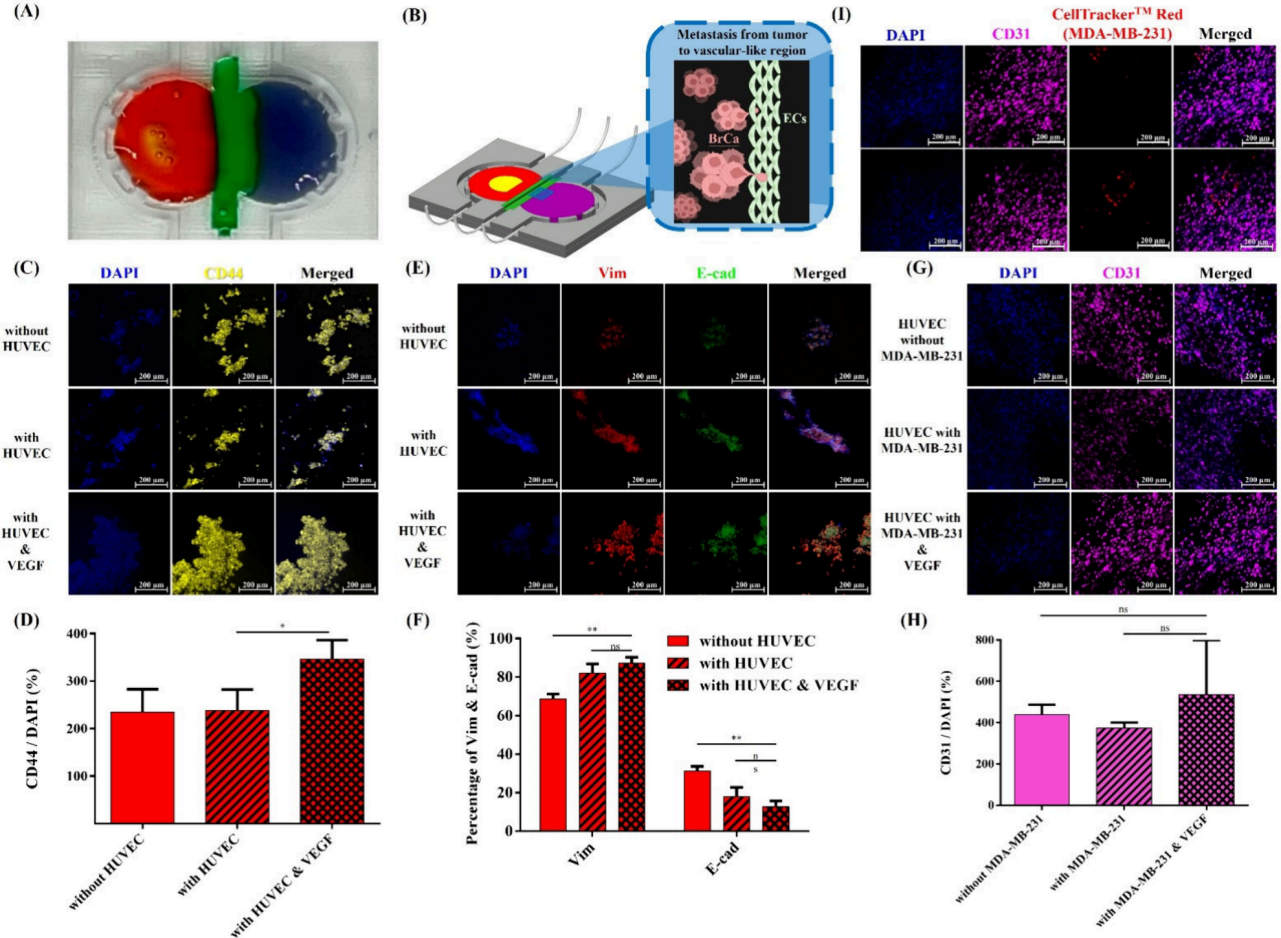
Intravasation of BrCa (MDA-MB-231) into the vascular-like region model. (A) 3D bioprinting of tumor, vascular-like region, and bone region on the chip. (B) Schematic diagram of BrCa cell intravasation into vascular-like region on a chip. Images of MDA-MB-231 intravasating into the vascular-like region with VEGF by using CellTracker Red. The CD44 expression of (C) immunofluorescence images and (D) quantification of MDA MB-231 in periphery hydrogels without HUVEC, with HUVEC, and with VEGF. The vimentin and E-cadherin expression of (E) immunofluorescence images and (F) quantification of MDA MB-231 in periphery hydrogels without HUVEC, with HUVEC, and with VEGF. The CD31 expression of (G) immunofluorescence images and (H) quantification of HUVEC in 1 wt % ColMA hydrogels without MDA-MB-231, with MDA-MB-231, and with VEGF. (I) Images of MDA-MB-231 intravasating into the vascular-like region by using CellTracker Red. Some components (e.g., cell spheroids) were created with BioRender.com, and the figure was further modified by the authors.

Figure 6(E) compares the expression of vimentin and E-cadherin in MDA-MB-231 cells cultured alone versus HUVEC-laden bioprinting for 7 days. Quantitative analysis presented in Figure 6(F) reveals that the vascular-like model with HUVECs significantly increases the expression of vimentin while decreasing E-cadherin levels, indicative of EMT and enhanced invasiveness of MDA-MB-231 cells. This finding is consistent with previous studies showing that ECs, including HUVECs, can promote EMT in cancer cells through the secretion of angiogenesis-related factors, such as TGF-β, VEGF, EGF, and PDGF, all of which are known to induce EMT and facilitate cancer cell migration and invasion.^30^ Upon the addition of VEGF, Figure 6(F) shows a slight increase in the level of vimentin expression, although the change was not statistically significant. This suggests that, although VEGF is known to promote invasiveness and enhance cancer stem cell-like properties in some tumor types, its effect on MDA-MB-231 cells may be limited under these experimental conditions. EMT is a dynamic process where epithelial cells transition to a mesenchymal phenotype, and most cells in the coculture had likely already completed EMT prior to VEGF addition. Consequently, the presence of VEGF had a minimal impact on vimentin expression. Furthermore, given that MDA-MB-231 cells are inherently mesenchymal, they may not show a marked response to VEGF compared to more epithelial-like cancer cells, a finding that is consistent with the literature showing limited VEGF-induced EMT in cells that are already in a mesenchymal state.

Quantitative analysis in Figure 6(H) shows that VEGF upregulates CD31 expression in HUVECs cocultured with MDA-MB-231 cells for 7 days. According to Jianqiang Wu et al., VEGF activates the MAPK/ERK pathway, disrupting intercellular adhesion and decreasing CD31 expression, which facilitates EC proliferation and migration during angiogenesis.^31^ The upregulation of CD31 in Figures 6(G) and (H) is likely due to reduced intercellular adhesion after VEGF addition, initiating HUVEC proliferation and migration while allowing MDA-MB-231 infiltration. After 7 days, intercellular adhesion increases, promoting angiogenesis and EC barrier formation, with CD31 expression returning to baseline levels, potentially due to VEGF’s role in enhancing EC involvement in angiogenesis. In addition, CellTracker Red was also used to track the infiltration of MDA-MB-231 cells into the vascular area under VEGF stimulation. As shown in Figure 6(I), a small number of MDA-MB-231 cells were observed to infiltrate the vascular-like region, successfully simulating the metastatic process. This observation demonstrates that the addition of VEGF enhances the metastatic potential of the MDA-MB-231 cells.

### 3.10 Bone Model Fabrication and Extravasation of BrCa into Bone Model on a Chip

The simulation of the skeletal microenvironment in ECM relies primarily on the selection of appropriate ECM components and the stiffness of the hydrogels. The skeletal ECM comprises approximately 70% inorganic components, predominantly HAp, and 30% organic components, primarily collagen. Bone, being a highly rigid tissue, has a Young’s modulus typically measured in the GPa range. However, hydrogels, as soft gel-like materials, cannot achieve such stiffness, limiting their ability to fully replicate the mechanical properties of bone.

To address the challenge of replicating the skeletal microenvironment, bioinks composed of 1 wt % ColMA and 2 wt % hydroxyapatite (HAp) were evaluated. As shown in Figure 7(A), this composition successfully formed hydrogels. However, the Young’s modulus of these hydrogels, illustrated in Figure 7(B), was reduced, likely due to the partial interference of HAp with ColMA cross-linking, resulting in incomplete polymerization and diminished mechanical stability. Based on previous literature,^32,33^ GelMA, a gelatin derivative, was selected to replace ColMA for improved bioink performance. Bioinks using 8 wt % GelMA alone, 8 wt % GelMA + 2 wt % HAp, and 8 wt % GelMA + 5 wt % HAp exhibited successful cross-linking, as shown in Figure 7(B). An increase in HAp content corresponded to an increase in Young’s modulus, with the highest stiffness observed in the 8 wt % GelMA + 5 wt % HAp formulation. This composition was selected as the optimal candidate due to its balance between mechanical properties and the solubility constraints of GelMA, which limited the preparation of higher concentrations. Further characterization of the 8 wt % GelMA + 5 wt % HAp bioink demonstrated its shear-thinning behavior, confirming its suitability for extrusion-based bioprinting (Figure S7­(A)). Additionally, swelling ratio analyses (Figure S7­(B)) indicated that this bioink composition maintained its structural integrity over time without significant swelling, ensuring compatibility with adjacent hydrogels. These findings position the 8 wt % GelMA/5 wt % HAp hydrogel as a robust platform for simulating the skeletal microenvironment.

**Figure 7:**
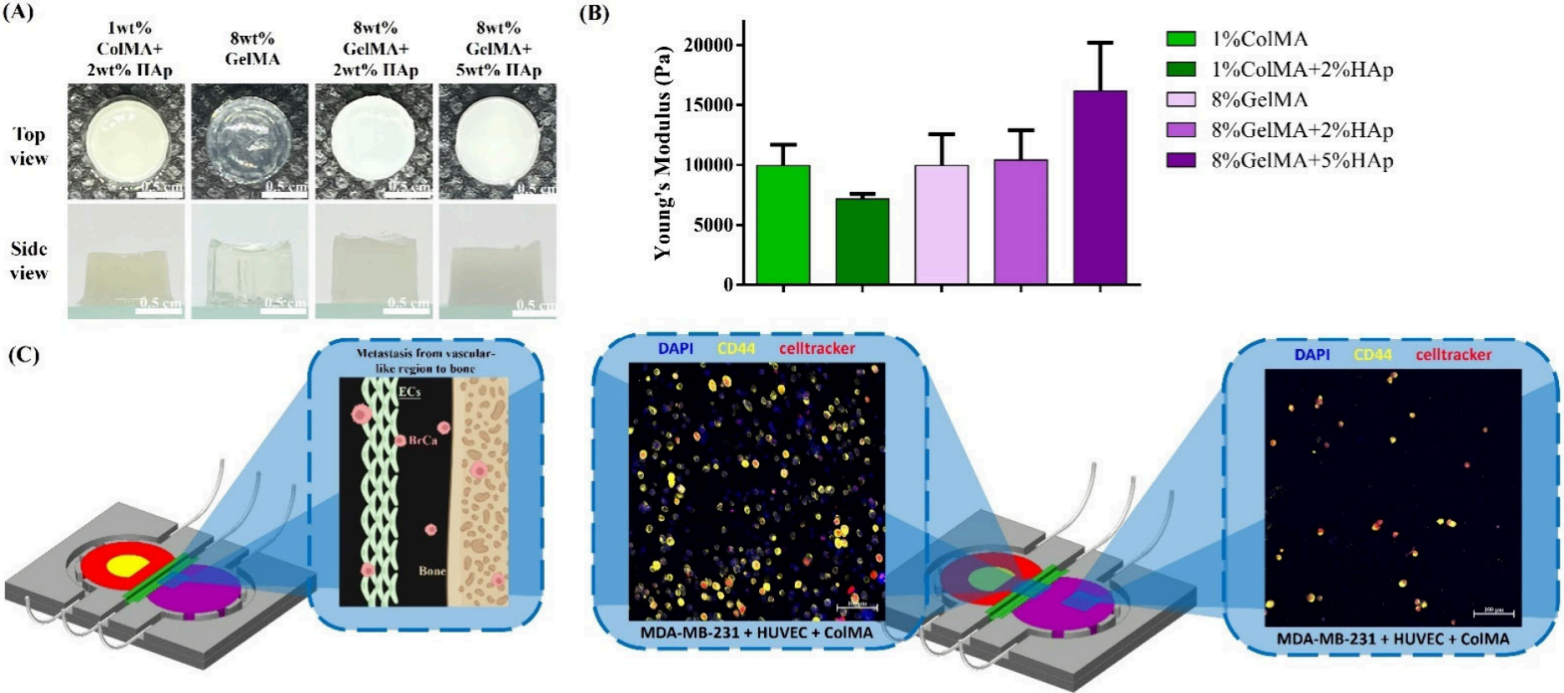
Extravasation of BrCa into bone model on a chip. (A) Top and side views of the ColMA/HAp and GelMA/HAp hydrogel morphology. (B) Young’s modulus of the ColMA/HAp and GelMA/HAp hydrogels. (C) Illustration of extravasation of BrCa into bone model and images of MDA-MB-231 extravasating from the vascular-like region into bone by using CellTracker Red. Some components (e.g., cell spheroids) were created with BioRender.com, and the figure was further modified by the authors.

Previous studies have demonstrated that BrCa cells extravasate from the vascular-like region, traversing the endothelial barrier to invade the bone microenvironment. To model this process, MDA-MB-231 cells were labeled with CellTracker Red and the CD44 marker to facilitate tracking. These labeled cells were subsequently incorporated into a 3D bioprinted vascular model on a microfluidic chip. Confocal microscopy was employed to monitor the cell migration dynamics. Figure 7(C) demonstrates that the highly invasive MDA-MB-231 cells successfully extravasated from the 3D-printed vascular model embedded with HUVECs into the bone-mimetic matrix composed of 8 wt % GelMA and 5 wt % HAp. Although some metastasis mechanisms remain unaddressed, this chip offers a robust platform for studying breast cancer metastasis *in vitro*. Future applications may include drug screening and personalized medicine, presenting a potential alternative to animal and clinical trials for evaluating therapeutic efficacy and patient-specific responses.

Our metastasis model provides a physiologically relevant platform for studying tumor–bone interactions. Preformed tumor spheroids replicate *in vivo* architecture, and tumor regions are engineered with heterogeneous stiffness to reflect the core–periphery gradient. The bone region incorporates hydroxyapatite (HAP), a major inorganic component of native bone ECM, enhancing hydrogel stiffness and more accurately reproducing bone mechanics. Independent perfusion of each region allows precise control of nutrient delivery, signaling molecule exchange and waste removal. Previous studies, such as Sukanya et al. (2024),^34^ generated bone models using native ECM-based hydrogels to reproduce biochemical cues, providing an excellent framework for studying cancer cell migration. While effective for biochemical fidelity, these ECM-based constructs often lack the mechanical stiffness and structural characteristics of native bone. By incorporation of HAP into our hydrogel, our model captures both the chemical and mechanical properties of bone tissue, offering a more comprehensive and physiologically relevant platform for investigating bone metastasis. Nevertheless, the study presented here has limitations. Only two breast cancer cell lines were used, and a limited set of hydrogel formulations were tested, which may not fully represent the heterogeneity of patient tumors. Additionally, although the hydrogel-based system allows precise control of mechanical and biochemical cues, it cannot completely recapitulate the complexity of native tissue architecture, including immune components and the dynamic remodeling of the bone microenvironment. Future refinements will integrate insights from ECM-based approaches and incorporate additional cell types or patient-derived samples to further enhance the physiological relevance and model fidelity.

## 4 Conclusion

This study developed a series of photopolymerizable HAMA and ColMA bioinks with shear-thinning properties and tunable mechanical characteristics, enabling the construction of 3D bioprinted models for studying BrCa metastasis. A tumor spheroid model revealed that the core bioink promoted BrCa cell proliferation, while the peripheral bioink enhanced migration and invasiveness via CD44 and EMT marker upregulation. A vascular model simulated BrCa cell intravasation, showing that HUVEC-facilitated EMT and MDA-MB-231 cells disrupted endothelial adhesion with VEGF, further promoting cancer stemness and invasiveness. The GelMA/HAp bioink effectively modeled the extravasation of invasive BrCa cells, demonstrating their migration from the vascular-like model into the bone–mimetic matrix. This system successfully recapitulated the final stages of the breast cancer metastasis. By integrating hydrogels, tumor spheroids, and microchannel systems, this study established an *in vitro* BrCa metastasis chip capable of replicating BrCa cell behaviors across the tumor core and periphery and during intravasation and extravasation. The chip also elucidated key interactions among MDA-MB-231 cells, HUVECs, and VEGF. This integrated 3D metastasis chip provides a valuable platform for studying BrCa progression and cell–microenvironment interactions, with potential applications in drug discovery and personalized medicine as an alternative to animal and clinical trials.

## Supplementary Material



## Data Availability

All data generated or analyzed during this study are included in this published article and its Supporting Information files.

## Notes

Table S1: Summary of the hydrogel formulations and their corresponding Young’s modulus used in this study. Figure S1. Chemical reaction formula, ^1^H NMR and FTIR spectrum of HAMA and ColMA. Figure S2: Biocompatibility of HAMA/ColMA/collagen hydrogels. Figure S3: Images of 3D tumor spheroid formation on different concentrations of PVA coating wells. Figure S4: Breast cancer spheroid formation. Figure S5: MCF-7 spheroids and SVEC4–10 on the chip. Figure S6: Representative images and quantitative analysis of the migration distance of MCF-7 and MDA-MB-231 cells in the core and peripheral regions at different culture times. Figure S7: Shear thinning and swelling ratio of GelMA+HAp bioink (PDF)

## References

[1] Sharma G. N., Dave R., Sanadya J., Sharma P., Sharma K. (2010). Various types
and management of breast cancer: an overview. Journal of advanced pharmaceutical technology & research.

[2] Kuchuk I., Hutton B., Moretto P., Ng T., Addison C. L., Clemons M. (2013). Incidence, consequences and treatment of bone metastases
in breast cancer patients—Experience from a single cancer centre. Journal of Bone Oncology.

[3] Place A. E., Jin Huh S., Polyak K. (2011). The microenvironment
in breast cancer
progression: biology and implications for treatment. Breast cancer research.

[4] Soysal S. D., Tzankov A., Muenst S. E. (2015). Role of the tumor microenvironment
in breast cancer. Pathobiology.

[5] Ananthanarayanan B., Kim Y., Kumar S. (2011). Elucidating the mechanobiology of malignant brain tumors
using a brain matrix-mimetic hyaluronic acid hydrogel platform. Biomaterials.

[6] Umesh V., Rape A. D., Ulrich T. A., Kumar S. (2014). Microenvironmental
stiffness enhances glioma cell proliferation by stimulating epidermal
growth factor receptor signaling. PLoS One.

[7] Fenner J., Stacer A. C., Winterroth F., Johnson T. D., Luker K. E., Luker G. D. (2014). Macroscopic stiffness
of breast tumors predicts metastasis. Sci. Rep.

[8] Chen J.-W. E., Pedron S., Shyu P., Hu Y., Sarkaria J. N., Harley B. A. C. (2018). Influence of Hyaluronic Acid Transitions
in Tumor Microenvironment
on Glioblastoma Malignancy and Invasive Behavior. Frontiers in Materials.

[9] Spearman B. S., Agrawal N. K., Rubiano A., Simmons C. S., Mobini S., Schmidt C. E. (2020). Tunable methacrylated hyaluronic acid-based hydrogels
as scaffolds for soft tissue engineering applications. J. Biomed. Mater. Res., Part A.

[10] Bourguignon L. Y., Wong G., Earle C., Chen L. (2012). Hyaluronan-CD44v3 interaction
with Oct4-Sox2-Nanog promotes miR-302 expression leading to self-renewal,
clonal formation, and cisplatin resistance in cancer stem cells from
head and neck squamous cell carcinoma. J. Biol.
Chem..

[11] Hiraga T., Ito S., Nakamura H. (2013). Cancer stem-like cell marker CD44 promotes bone metastases
by enhancing tumorigenicity, cell motility, and hyaluronan production. Cancer research.

[12] Plodinec M., Loparic M., Monnier C. A., Obermann E. C., Zanetti-Dallenbach R., Oertle P., Hyotyla J. T., Aebi U., Bentires-Alj M., Lim R. Y. (2012). The
nanomechanical signature of breast cancer. Nature
Nanotechnol..

[13] Ahmad A., Nawaz M. I. (2022). Molecular mechanism of VEGF and its role in pathological
angiogenesis. Journal of Cellular Biochemistry.

[14] Abudupataer M., Chen N., Yan S., Alam F., Shi Y., Wang L., Lai H., Li J., Zhu K., Wang C. (2020). Bioprinting a 3D vascular construct
for engineering a vessel-on-a-chip. Biomed.
Microdevices.

[15] Wang D., Maharjan S., Kuang X., Wang Z., Mille L. S., Tao M., Yu P., Cao X., Lian L., Lv L. (2022). Microfluidic bioprinting of tough hydrogel-based vascular conduits
for functional blood vessels. Science Advances.

[16] Bahcecioglu G., Basara G., Ellis B. W., Ren X., Zorlutuna P. (2020). Breast cancer
models: Engineering the tumor microenvironment. Acta biomaterialia.

[17] Ravi M., Paramesh V., Kaviya S. R., Anuradha E., Solomon F. D. (2015). 3D cell
culture systems: advantages and applications. J. Cell Physiol.

[18] Datta P., Dey M., Ataie Z., Unutmaz D., Ozbolat I. T. (2020). 3D bioprinting for
reconstituting the cancer microenvironment. npj Precision Oncology.

[19] Wang Y., Shi W., Kuss M., Mirza S., Qi D., Krasnoslobodtsev A., Zeng J., Band H., Band V., Duan B. (2018). 3D Bioprinting
of Breast Cancer Models for Drug Resistance Study. ACS Biomaterials Science & Engineering.

[20] Belgodere J. A., King C. T., Bursavich J. B., Burow M. E., Martin E. C., Jung J. P. (2018). Engineering Breast
Cancer Microenvironments and 3D
Bioprinting. Frontiers in Bioengineering and
Biotechnology.

[21] Meng F., Meyer C. M., Joung D., Vallera D. A., McAlpine M. C., Panoskaltsis-Mortari A. (2019). 3D Bioprinted In Vitro Metastatic Models via Reconstruction
of Tumor Microenvironments. Adv. Mater..

[22] Zhou X., Zhu W., Nowicki M., Miao S., Cui H., Holmes B., Glazer R. I., Zhang L. G. (2016). 3D Bioprinting a Cell-Laden Bone
Matrix for Breast Cancer Metastasis Study. ACS
Appl. Mater. Interfaces.

[23] Velasco-Rodriguez B., Diaz-Vidal T., Rosales-Rivera L. C., García-González C. A., Alvarez-Lorenzo C., Al-Modlej A., Domínguez-Arca V., Prieto G., Barbosa S., Soltero Martínez J. F. A. (2021). Hybrid methacrylated gelatin and hyaluronic acid hydrogel
scaffolds. Preparation and systematic characterization for prospective
tissue engineering applications. International
Journal of Molecular Sciences.

[24] Naomi R., Ridzuan P. M., Bahari H. (2021). Current insights into collagen type
I. Polymers.

[25] Xia C., Chen P., Mei S., Ning L., Lei C., Wang J., Zhang J., Ma J., Fan S. (2017). Photo-crosslinked
HAMA hydrogel with cordycepin encapsulated chitosan microspheres for
osteoarthritis treatment. Oncotarget.

[26] Song X., Dong P., Gravesande J., Cheng B., Xing J. (2018). UV-mediated
solid-state cross-linking of electrospinning nanofibers of modified
collagen. Int. J. Biol. Macromol..

[27] Zhang Q., Tang Q., Yang Y., Yi J., Wei W., Hong Y., Zhang X., Zhou F., Yao X., Ouyang H. (2021). Wound dressing gel with resisted bacterial penetration
and enhanced re-epithelization for corneal epithelial-stromal regeneration. Applied Materials Today.

[28] Pickup M. W., Mouw J. K., Weaver V. M. (2014). The extracellular
matrix modulates
the hallmarks of cancer. EMBO Rep.

[29] Insua-Rodríguez J., Oskarsson T. (2016). The extracellular matrix in breast cancer. Adv. Drug Deliv Rev..

[30] Wang L., Zhao L., Zhang L., Jing X., Zhang Y., Shao S., Zhao X., Luo M. (2021). [Vascular
endothelial
growth factor promotes cancer stemness of triple-negative breast cancer
via MAPK/ERK pathway]. Nan Fang Yi Ke Da Xue
Xue Bao.

[31] Wu J., Sheibani N. (2003). Modulation of VE-cadherin and PECAM-1 mediated cell-cell
adhesions by mitogen-activated protein kinases. J. Cell Biochem.

[32] Pu X., Tong L., Wang X., Liu Q., Chen M., Li X., Lu G., Lan W., Li Q., Liang J. (2022). Bioinspired hydrogel anchoring 3DP GelMA/HAp scaffolds accelerates
bone reconstruction. ACS Appl. Mater. Interfaces.

[33] Song P., Li M., Zhang B., Gui X., Han Y., Wang L., Zhou W., Guo L., Zhang Z., Li Z. (2022). DLP fabricating of precision GelMA/HAp porous composite scaffold
for bone tissue engineering application. Composites
Part B: Engineering.

[34] Sukanya V. S., Mehta V., Jilla S., Rath S. N. (2024). Differential osteo-specific
invasion of patient-derived cancer cells in a microfluidic co-culture
model. Chemical Engineering Journal.

