# Supplementary material for: Construction of a 3D Bioprinted Microfluidic Platform to Study Breast Cancer Bone Metastasis and Tumor Microenvironmental Influences
Source: ACS Appl Mater Interfaces. 2025 Oct 31;17(45):61718–30. doi: 10.1021/acsami.5c15529 (PMC12616590; doi:10.1021/acsami.5c15529)
Supplement: Supplementary file 1 [file am5c15529_si_001.pdf]

Supporting Information

Construction of a 3D Bioprinted Microfluidic Platform to Study Breast Cancer Bone Metastasis and Tumor Microenvironmental Influences

Ting-Wei Chang [1], Min-Wei Huang [1], Guo-Chung Dong [2, #], and I-Chi Lee [1, *]

[1] Department of Biomedical Engineering and Environmental Sciences, National Tsing Hua University, No. 101, Section 2, Kuang-Fu Road, Hsinchu 300044, Taiwan (R.O.C)

[2]Institute of Biomedical Engineering and Nanomedicine, National Health Research Institutes, No.35, Keyan Rd., Zhunan Town, Miaoli 35053, Taiwan (R.O.C)

* Corresponding author: I-Chi Lee

Mailing address: 300044 No. 101, Section 2, Kuang-Fu Road, Hsinchu, Taiwan, ROC

E-mail: iclee@mx.nthu.edu.tw

Tel: +886-3-5715131 ext 35525

# Co-Corresponding author: Guo-Chung Dong

Email: gcdong@nhri.org.tw

Table S1. Summary of the hydrogel formulations and their corresponding Young's modulus (mean± standard deviation) used in this study. Three representative formulations were selected to model distinct tumor microenvironments: softer hydrogels to mimic the tumor core, stiffer hydrogels to approximate the tumor periphery, and ColMA-based formulation was employed to represent the vascular-like region.

**Hydrogel composition**			**Young's modulus (kPa, mean ± SD)**		**Assigned region in model**
HAMA (%)	ColMA (%)	Collagen (%)	average	standard deviation	
1.5	0	1	1733.90	400.43	Tumor core
1.5	0	3.5	2600.85	346.78	
2	0	2	4161.36	800.85	
3	0	0	6935.60	566.29	
3	0	1	10403.40	2402.56	Tumor periphery
0	1	0	9709.84	1498.26	Vascular-like region
1	1	1	2427.46	400.43	
2	1	1	3121.02	400.43	
0	1.5	0	11096.96	1878.17	

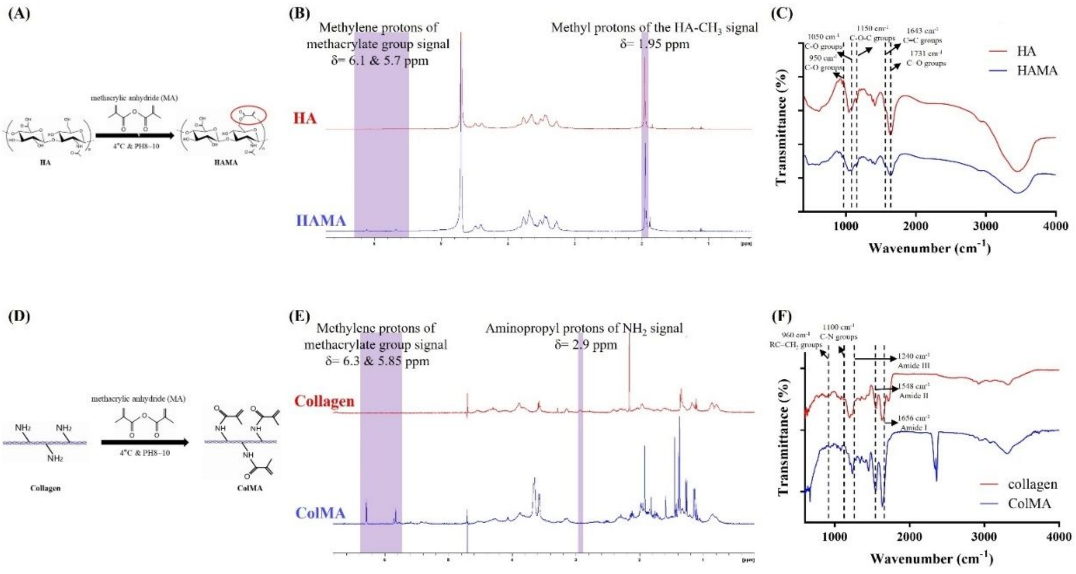

Figure S1. Chemical reaction formula of (A) HAMA and (D) ColMA. (B) ^{1}H-NMR spectrum of HA and HAMA. (C) FTIR spectrum of HA and HAMA. (E) ^{1}H-NMR spectrum of collagen and ColMA. (F) FTIR spectrum of collagen and ColMA.

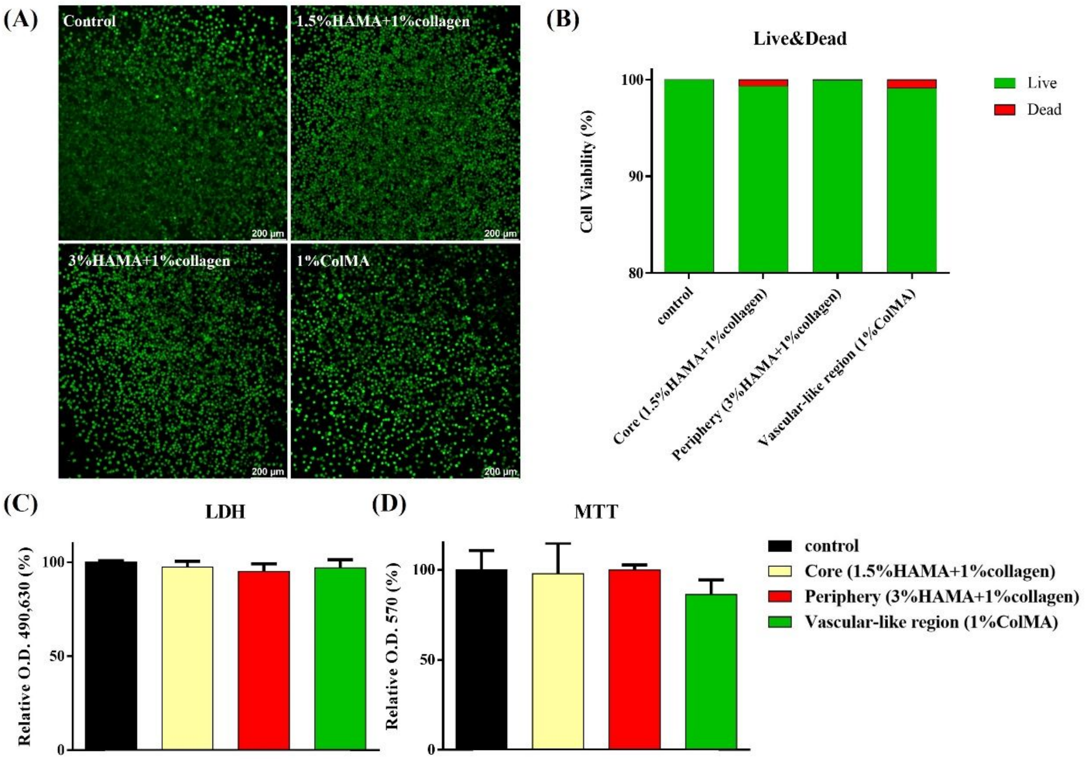

Figure S2. Biocompatibility of HAMA/ColMA/collagen hydrogels. (A) Image of Live/Dead Staining of L929 Cells (B) Quantification of Live/Dead Staining, (C) LDH assay, and (D) MTT assay.

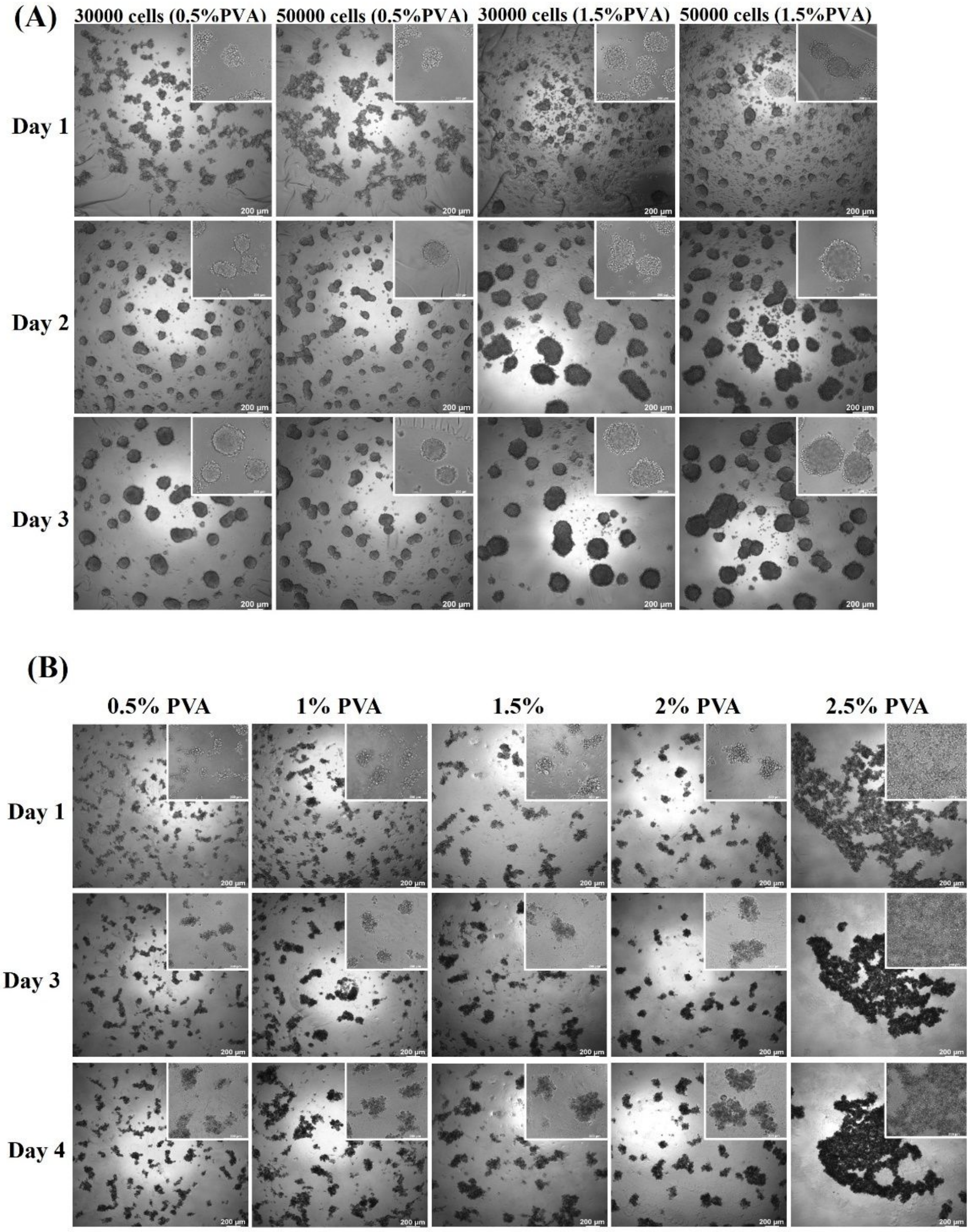

Figure S3. Images of 3D tumor spheroids formation of (A) MCF-7 at day 1, 2 and 3 and (B) MDA-MB-231 at day 1, 3 and 4 on different concentration of PVA coating well

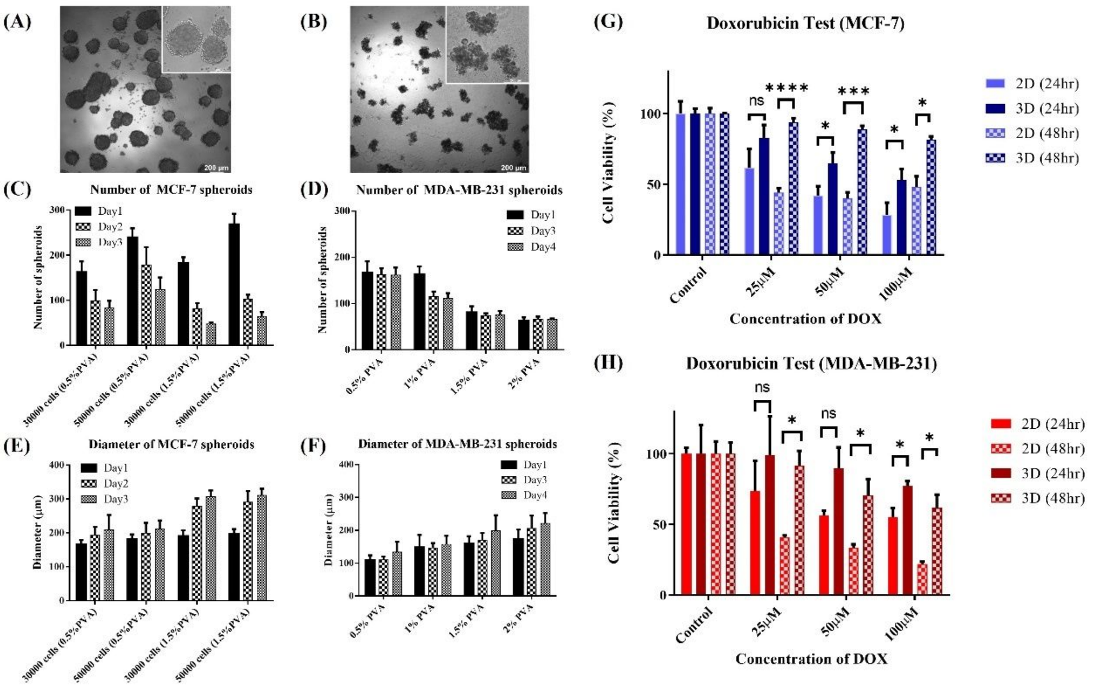

Figure S4. Formation and characterization of breast cancer spheroids under optimized PVA coating conditions. Representative images of 3D tumor spheroids of (A) MCF-7 at day 3 with 1.5 wt% PVA and (B) MDA-MB-231 at day 4 with 2 wt% PVA. Quantification of spheroid number per well for (C) MCF-7 and (D) MDA-MB-231. Diameter measurements of spheroids for (E) MCF-7 and (F) MDA-MB-231. Cell viability of 3D spheroids compared to 2D culture in response to different Doxorubicin concentrations for (G) MCF-7 and (H) MDA-MB-231 at 24 and 48 hours. Statistical significance is denoted by asterisks as follows: *p<0.05, **p<0.01, ***p<0.005, and ****p<0.001.

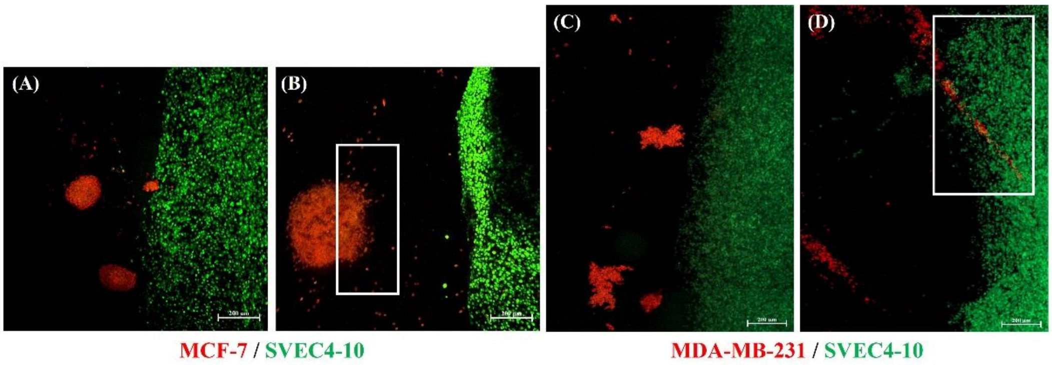

Figure S5. MCF-7 spheroids (CellTracker™ Red) and SVEC4-10 (CellTracker™ Green) on the chip at (A) day 0 and (B) day 7. MDA-MB-231 spheroids (CellTracker™ Red) and SVEC4-10 (CellTracker™ Green) on the chip at (C) day 0 and (D) day 7.

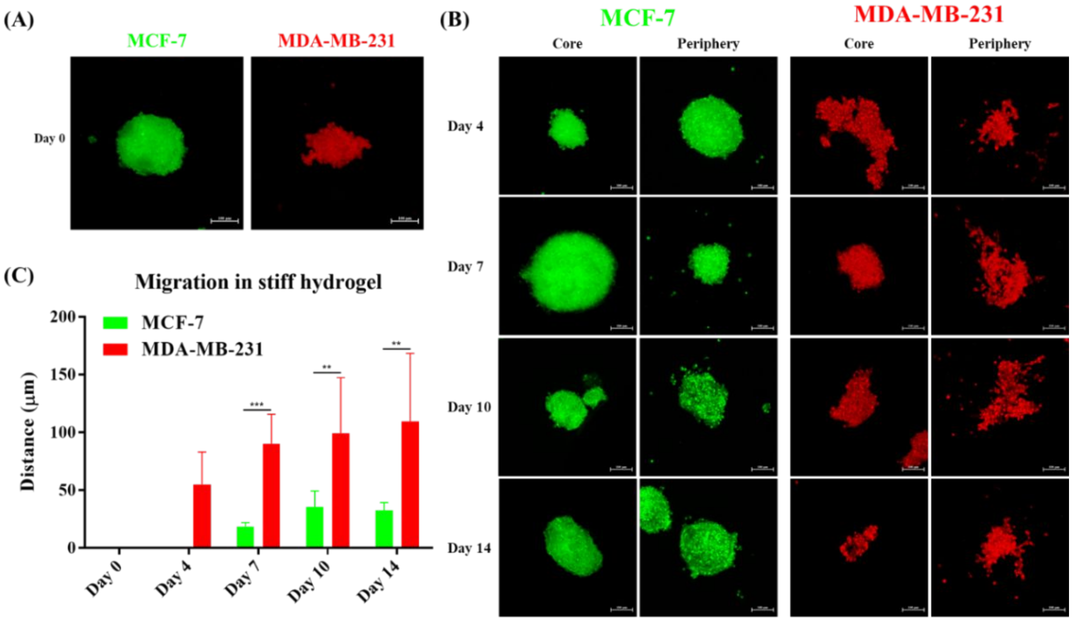

Figure S6. (A) 3D BrCa spheroids at day 0 without hydrogel embedding. (B) Representative images showing cell migration from MCF-7 and MDA-MB-231 spheroids within core and peripheral hydrogels. (C) Quantification of migration distance of MCF-7 and MDA-MB-231 cells in core and peripheral regions at days 4, 7, 10, and 14. Statistical significance is denoted by asterisks as follows: *$p<0.05$, **$p<0.01$, ***$p<0.005$, and ****$p<0.001$.

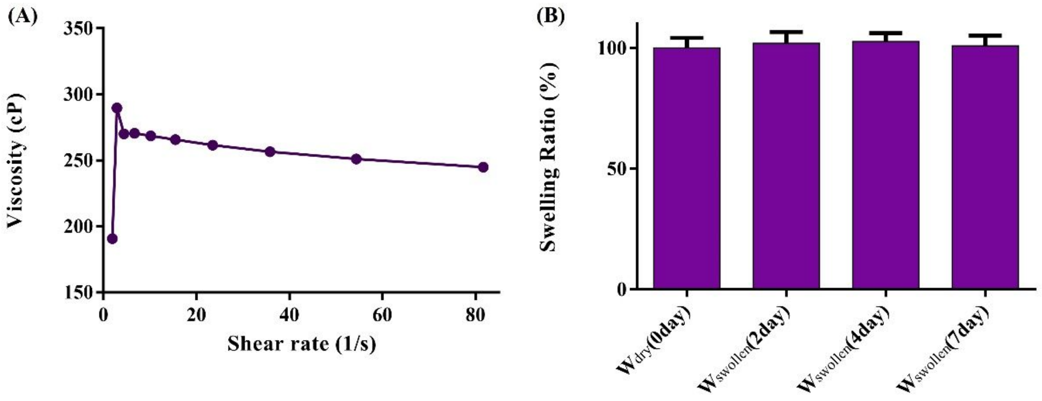

Figure S7. (A) Shear thinning of 8wt% GelMA + 5wt% HAp bioinks. (B) Swelling ratio of 8wt% GelMA + 5wt% HAp hydrogel.
